# The *Staphylococcus aureus* Global Regulator MgrA Modulates Clumping and Virulence by Controlling Surface Protein Expression

**DOI:** 10.1371/journal.ppat.1005604

**Published:** 2016-05-04

**Authors:** Heidi A. Crosby, Patrick M. Schlievert, Joseph A. Merriman, Jessica M. King, Wilmara Salgado-Pabón, Alexander R. Horswill

**Affiliations:** Department of Microbiology, Roy J. and Lucille A. Carver College of Medicine, University of Iowa, Iowa City, Iowa, United States of America; National Institutes of Health, UNITED STATES

## Abstract

*Staphylococcus aureus* is a human commensal and opportunistic pathogen that causes devastating infections in a wide range of locations within the body. One of the defining characteristics of *S*. *aureus* is its ability to form clumps in the presence of soluble fibrinogen, which likely has a protective benefit and facilitates adhesion to host tissue. We have previously shown that the ArlRS two-component regulatory system controls clumping, in part by repressing production of the large surface protein Ebh. In this work we show that ArlRS does not directly regulate Ebh, but instead ArlRS activates expression of the global regulator MgrA. Strains lacking *mgrA* fail to clump in the presence of fibrinogen, and clumping can be restored to an *arlRS* mutant by overexpressing either *arlRS* or *mgrA*, indicating that ArlRS and MgrA constitute a regulatory pathway. We used RNA-seq to show that MgrA represses *ebh*, as well as seven cell wall-associated proteins (SraP, Spa, FnbB, SasG, SasC, FmtB, and SdrD). EMSA analysis showed that MgrA directly represses expression of *ebh* and *sraP*. Clumping can be restored to an *mgrA* mutant by deleting the genes for Ebh, SraP and SasG, suggesting that increased expression of these proteins blocks clumping by steric hindrance. We show that *mgrA* mutants are less virulent in a rabbit model of endocarditis, and virulence can be partially restored by deleting the genes for the surface proteins *ebh*, *sraP*, and *sasG*. While *mgrA* mutants are unable to clump, they are known to have enhanced biofilm capacity. We demonstrate that this increase in biofilm formation is partially due to up-regulation of SasG, a surface protein known to promote intercellular interactions. These results confirm that ArlRS and MgrA constitute a regulatory cascade, and that they control expression of a number of genes important for virulence, including those for eight large surface proteins.

## Author Summary

*Staphylococcus* causes a wide range of diseases, ranging from skin infections to deadly invasive condition like endocarditis, septicemia, osteomyelitis, and pneumonia. In this work we examine the ArlRS two-component regulatory system, which controls interactions with the host plasma protein fibrinogen. *S*. *aureus* normally forms large aggregates called clumps in the presence of fibrinogen, but the *arlRS* mutant is unable to clump. We demonstrate that ArlRS activates expression of the DNA-binding protein MgrA, and that *mgrA* is also required for clumping. Transcriptional analysis of an *mgrA* mutant shows that MgrA regulates expression of eight surface proteins. Expression of these surface proteins affects clumping, possibly by physically interfering with fibrinogen binding. Strains lacking *mgrA* are less virulent in an endocarditis model, and virulence can be partially restored by deleting genes for three of these surface proteins. An *mgrA* mutant is also known to have enhanced biofilm formation, and we show that this is partially due to increased production of one of these surface proteins. These results demonstrate that ArlRS and MgrA constitute a regulatory cascade in *S*. *aureus* that is crucial for pathogenesis and may be a good candidate to target for drug development.

## Introduction

*Staphylococcus aureus* is a human commensal that asymptomatically colonizes the nares, throat, and skin of ~30% of the population [1,2]. It is also a pervasive opportunistic pathogen that is the most common infectious agent isolated from hospital inpatients in the US [3]. *S*. *aureus* causes a range of diseases, from skin and soft tissue infections to life-threatening conditions like pneumonia, osteomyelitis, sepsis and infective endocarditis. Antibiotic resistance has been increasing among *S*. *aureus* isolates in the past few decades [4], limiting the available treatment options. For example, invasive infections caused by methicillin-resistant *S*. *aureus* (MRSA) have mortality rates approaching 20% [5], highlighting the need for innovative therapies.

*S*. *aureus* strains encode a wide variety of virulence factors, including up to 24 different cell wall anchored proteins that are covalently attached to the peptidoglycan layer by the transpeptidase sortase [6]. A subset of these, termed Microbial Surface Components Recognizing Adhesive Matrix Molecules (MSCRAMMs), are critical for attaching to components of the host extracellular matrix, such as fibrinogen, fibronectin, and collagen. Two of these MSCRAMMs, Clumping Factors A and B (ClfA and ClfB), facilitate *S*. aureus binding to fibrinogen and lead to agglutination or clumping of cells [7–10], and for simplicity, we will use the term clumping throughout. Fibrinogen is an abundant, soluble, 340 kDa elongated glycoprotein present in plasma that is processed by thrombin to form insoluble fibrin clots, a property that is crucial for blood clotting and platelet aggregation. *S*. *aureus* facilitates this conversion of fibrinogen to fibrin by secreting two coagulases that activate prothrombin, coagulase (Coa) and von Willibrand factor binding protein (vWbp). Notably ClfA can interact with both soluble fibrinogen and fibrin cables with similar affinities [11].

Clumping is thought to have a number of functions in the context of staphylococcal infections. Clumps are likely to be more resistant to clearance by the immune system, in part because they may be too large to be phagocytosed by neutrophils [12]. In addition, the fibrinogen coating on cells within a clump may impede antibody binding and complement deposition [13–15]. *S*. *aureus* can also form similar aggregates in the presence of synovial fluid that are dependent on ClfA/ClfB binding to fibrin [16], and these aggregates appear to be more resistant to antibiotic treatment, analogous to the recalcitrance to antibiotics seen with surface-attached biofilms [16]. Finally, it has been demonstrated that the cell density-dependent *agr* quorum sensing system, which controls expression of many virulence factors, is turned on in clumps, likely due to the increased local concentration of autoinducing peptide [17].

Clumping factors A and B are necessary for forming clumps and play an essential role in pathogenesis. Their genes are differentially regulated, with *clfB* being primarily expressed during exponential phase [18,19], and *clfA* expression increasing in later growth stages [20]. Strains lacking both *clfA* and *clfB* are unable to bind to fibrinogen, and because of this they fail to clump with fibrin and platelets *in vitro* [21]. Adhesion to platelets and fibrin is particularly important in the growth of vegetations on heart valves at the onset of infective endocarditis. These vegetations consist of bacteria, platelets, and fibrin, and, as expected, strains lacking *clfA* are less virulent in a rat model of endocarditis [22]. In addition, expressing *clfA* exogenously in normally nonpathogenic *Lactococcus lactis* significantly enhances its ability to generate heart valve vegetations [23]. *clfA* mutants also cause fewer septic arthritis symptoms [24] and are less lethal in bacteremia models [11,24–26]. In support of this, mice engineered to express a modified version of fibrinogen lacking the ClfA binding site are less susceptible to *S*. *aureus* bacteremia [27].

Until recently, clumping was assumed to be a passive property of *S*. *aureus* that was not subject to regulation. Yet other virulence factors of *S*. *aureus* are highly regulated, suggesting that clumping may also be modulated in response to environmental cues. *S*. *aureus* has 16 two-component systems (TCS) that respond to environmental signals and alter transcription accordingly [28], and these systems typically consist of a membrane-bound histidine kinase sensor protein and a DNA-binding response regulator. One of these TCSs is the *agr* quorum sensing system, which regulates a large number of virulence related secreted products, including cytolysins, proteases, phenol-soluble modulins, lipases, and superantigens [29]. Likewise, the SaeRS TCS regulates expression of a variety of virulence-related secreted proteins, such as the coagulases, hemolysins, and matrix binding proteins [30]. A third TCS, ArlRS, has been linked to virulence [31–33], but there is still relatively little known about which gene(s) it regulates and what signal activates the system. We have previously demonstrated that an *arlRS* mutant has a clumping defect and that it is attenuated in a rabbit model of endocarditis [33]. This failure to clump appears to be due, in part, to overproduction of the large surface protein Ebh, which may interfere with fibrinogen binding through steric hindrance [33]. Ebh, also known as the Giant Staphylococcal Surface Protein (GSSP), is an ~1.1 MDa protein of unknown function anchored at its C terminus in the cell membrane [34,35]. In this work we show that ArlR regulates Ebh production indirectly; we demonstrate that ArlR activates expression of the global regulator MgrA, which in turn represses *ebh*. We used RNA-seq to identify genes regulated by MgrA in USA300 strain LAC, and found eight genes for surface proteins that are repressed by MgrA, including *ebh*. We show that *mgrA* mutants are unable to clump, and that clumping can be restored by also deleting genes for the surface proteins Ebh and SraP in strain LAC. These results indicate that ArlRS and MgrA constitute a regulatory cascade that controls expression of a large number of genes, including those for Ebh and seven cell wall anchored proteins.

## Results

### MgrA is required for clumping

We have previously shown that the ArlRS TCS is required for *S*. *aureus* clumping, and that this is due in part to ArlRS suppressing production of the large surface protein Ebh [33]. However, we were unable to show direct binding of purified ArlR to the *ebh* promoter, despite several attempts with electrophoretic mobility shift assays (EMSAs), with unphosphorylated or phosphorylated ArlR. This led us to hypothesize that ArlRS modulates expression of *ebh* indirectly, presumably through the action of another regulator. In support of this idea, ArlRS was previously shown to regulate capsule expression indirectly by up-regulating expression of the global regulator MgrA [36]. MgrA modulates >10% of the *S*. *aureus* genome, and indeed one of largest differences in gene expression was the upregulation of *ebh* in an *mgrA* mutant [37].

**Fig 1:**
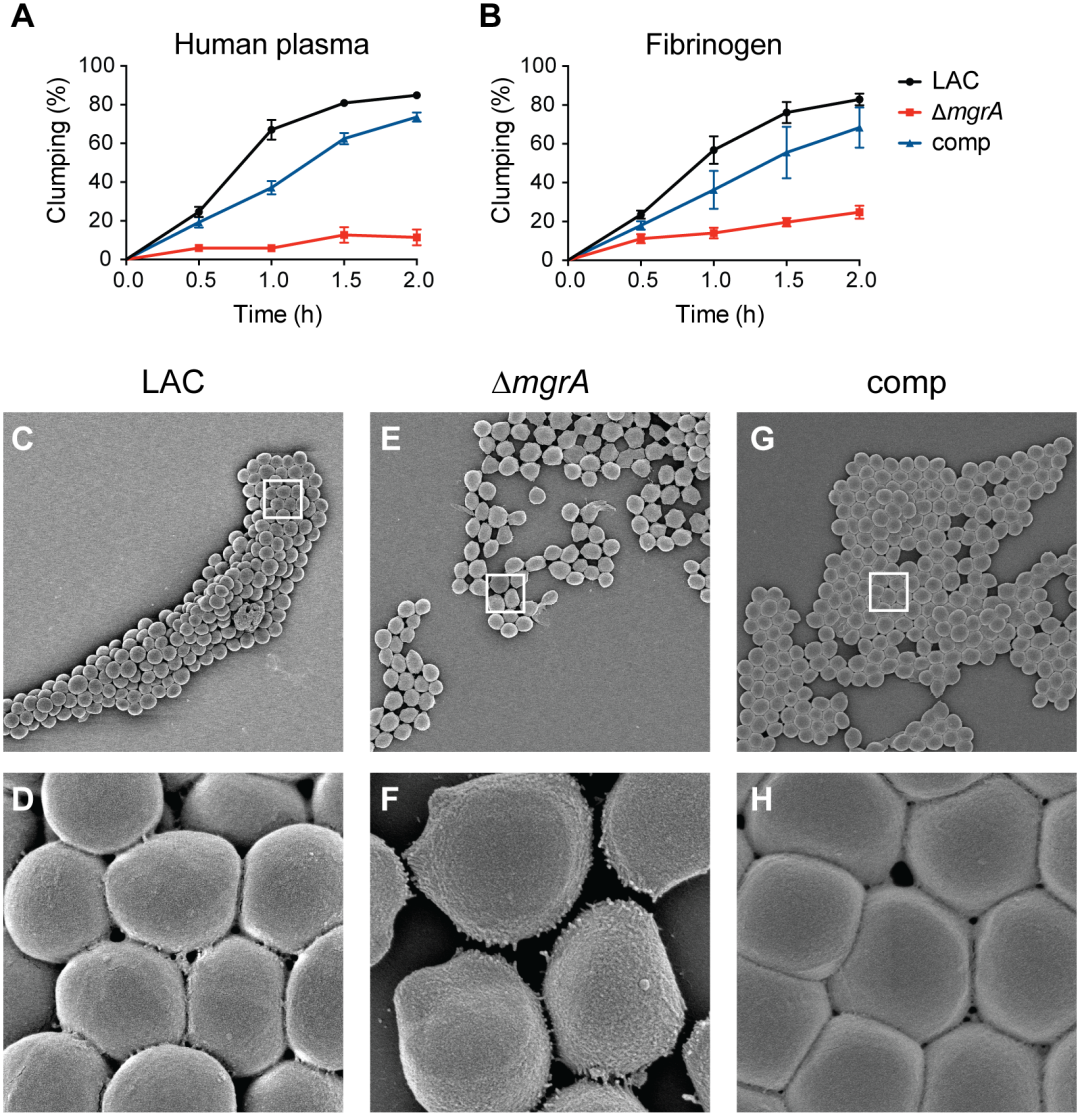
MgrA is required for *S*. *aureus* clumping. Washed *S*. *aureus* cells were incubated statically with either human plasma (A) or fibrinogen (B) and clumping was monitored by measuring clearing of the solution over time, as described in the methods section. Strains tested were the wild type USA300 LAC (black), an isogenic *mgrA* deletion (red), and the *mgrA* mutant complemented with a copy of *mgrA* expressed from an exogenous site on the chromosome (blue). Values represent averages and standard deviations of three separate experiments. Scanning electron micrographs of the same strains after incubation with fibrinogen for 2 h are shown in panels C-H. The white boxes in the top panels show the region magnified in the lower panels. Magnification is 5,000X in panels C, E, and G, and 45,000X in panels D, F, and H.

*S*. *aureus* strains lacking *mgrA* show increased autolysis [38] and biofilm formation [39], but MgrA has not been linked to clumping. We constructed an *mgrA* deletion in the USA300 strain LAC. Compared to USA300 WT, the *mgrA* mutant had a pronounced clumping defect with both human plasma (Fig 1A) and purified human fibrinogen (Fig 1B). This defect could be partially complemented by expressing *mgrA* under the control of its own promoters at the phage 11 integration site on the chromosome (Fig 1A and 1B), or by expression from a plasmid (used in epistasis studies shown below). Scanning electron microscopy (SEM) images of the wild type and Δ*mgrA* mutant after incubation with fibrinogen show a distinct difference in cell packing that can be complemented (Fig 1C–1H). It is somewhat surprising that *mgrA* mutants have a clumping defect, given their increased capacity to form a biofilm [39,40].

### ArlRS and MgrA constitute a regulatory pathway

**Fig 2:**
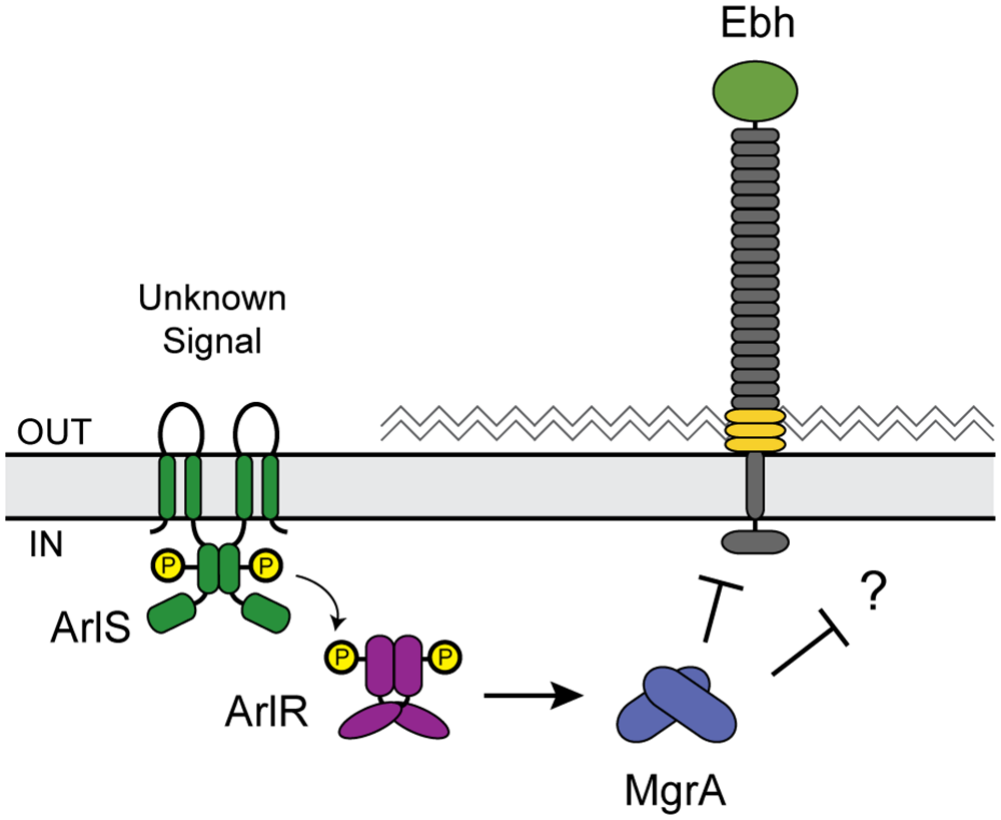
Model of how the ArlRS two-component system and MgrA control clumping. In response to an unknown signal ArlS either phosphorylates or dephosphorylates ArlR. When ArlR is phosphorylated it binds to the *mgrA* promoter, activating expression of *mgrA*. MgrA in turn represses *ebh* and other unknown targets. When either *arlRS* or *mgrA* is disrupted then *ebh* is de-repressed, and production of Ebh presumably blocks clumping.

**Fig 3:**
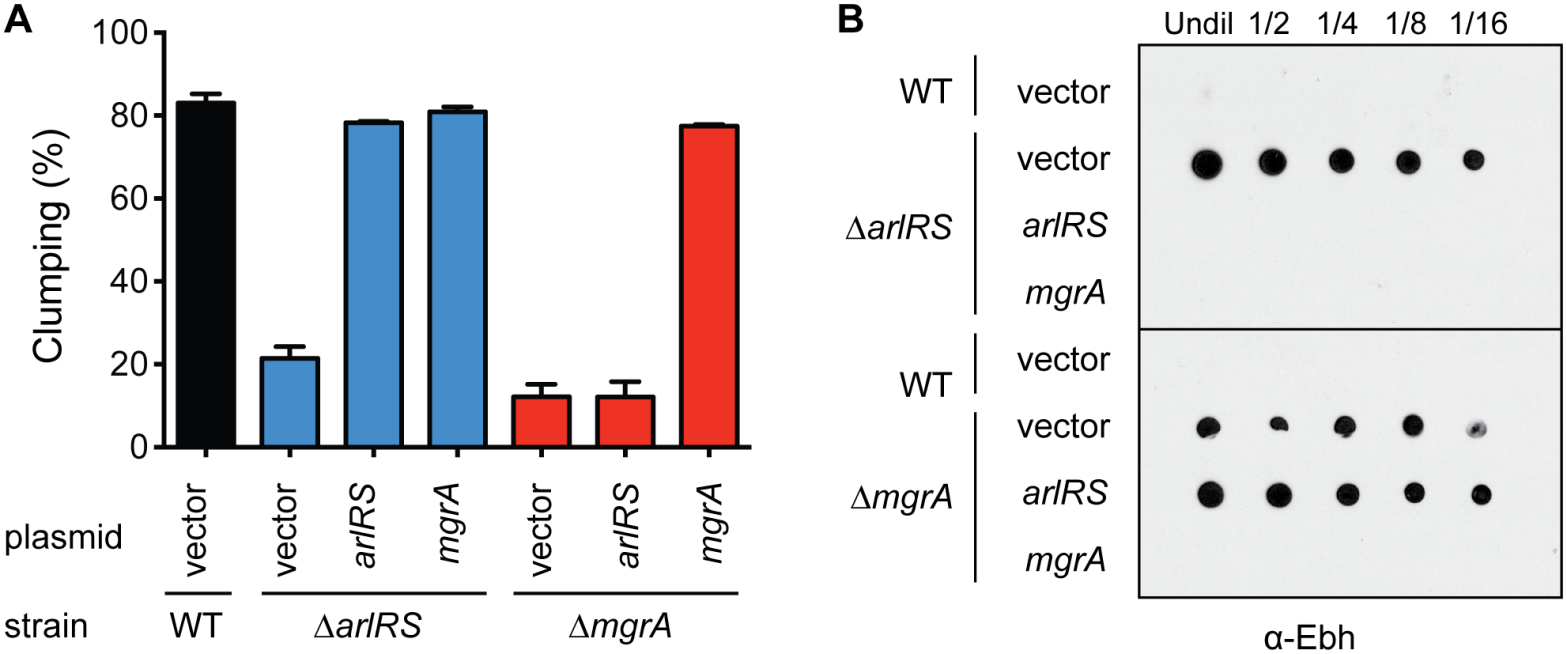
Epistasis analysis indicates MgrA acts downstream of ArlRS. LAC WT, *arlRS*, and *mgrA* mutants contained either the empty vector or plasmids for constitutive expression of *arlRS* or *mgrA*. (A) Clumping ability was measured after two hours of incubation with human plasma, and results from three separate experiments were averaged. (B) Dot blot showing Ebh protein levels in 2-fold dilutions of culture supernatants from overnight cultures. Strains were identical to those used in Fig 3A except that they lacked *spa*, to prevent non-specific antibody binding.

The similar clumping defects of *arlRS* and *mgrA* mutants led us to propose a model in which the ArlRS TCS activates expression of MgrA (Fig 2). MgrA in turn represses *ebh* and possibly other clumping-related genes, allowing the wild type strain to interact with fibrinogen and clump. There is evidence that MgrA and ArlRS work together, although the exact mechanism is unclear. It was initially reported that MgrA controlled *arlRS* expression [38], but a later study used qRT-PCR to show that ArlRS regulated MgrA [36]. To assess whether ArlRS acts upstream of MgrA as depicted in our model (Fig 2), we tested if overexpressing *mgrA* could complement an *arlRS* mutant. Indeed, the expression of *mgrA* from a multicopy plasmid restored clumping to an *arlRS* mutant, but expressing *arlRS* in an *mgrA* mutant had no effect (Fig 3A). As an additional epistasis test, we looked at production of Ebh, which is elevated in *arlRS* and *mgrA* mutant strains. Ebh could be restored to wild type levels in an *arlRS* mutant by expressing either *arlRS* or *mgrA* (Fig 3B). Similar to the clumping results, expressing *arlRS* did not restore Ebh production to an *mgrA* mutant (Fig 3B). These results demonstrate that ArlRS and MgrA form a regulatory cascade in which MgrA acts downstream of ArlRS.

### The ArlRS two-component system regulates MgrA

**Fig 4:**
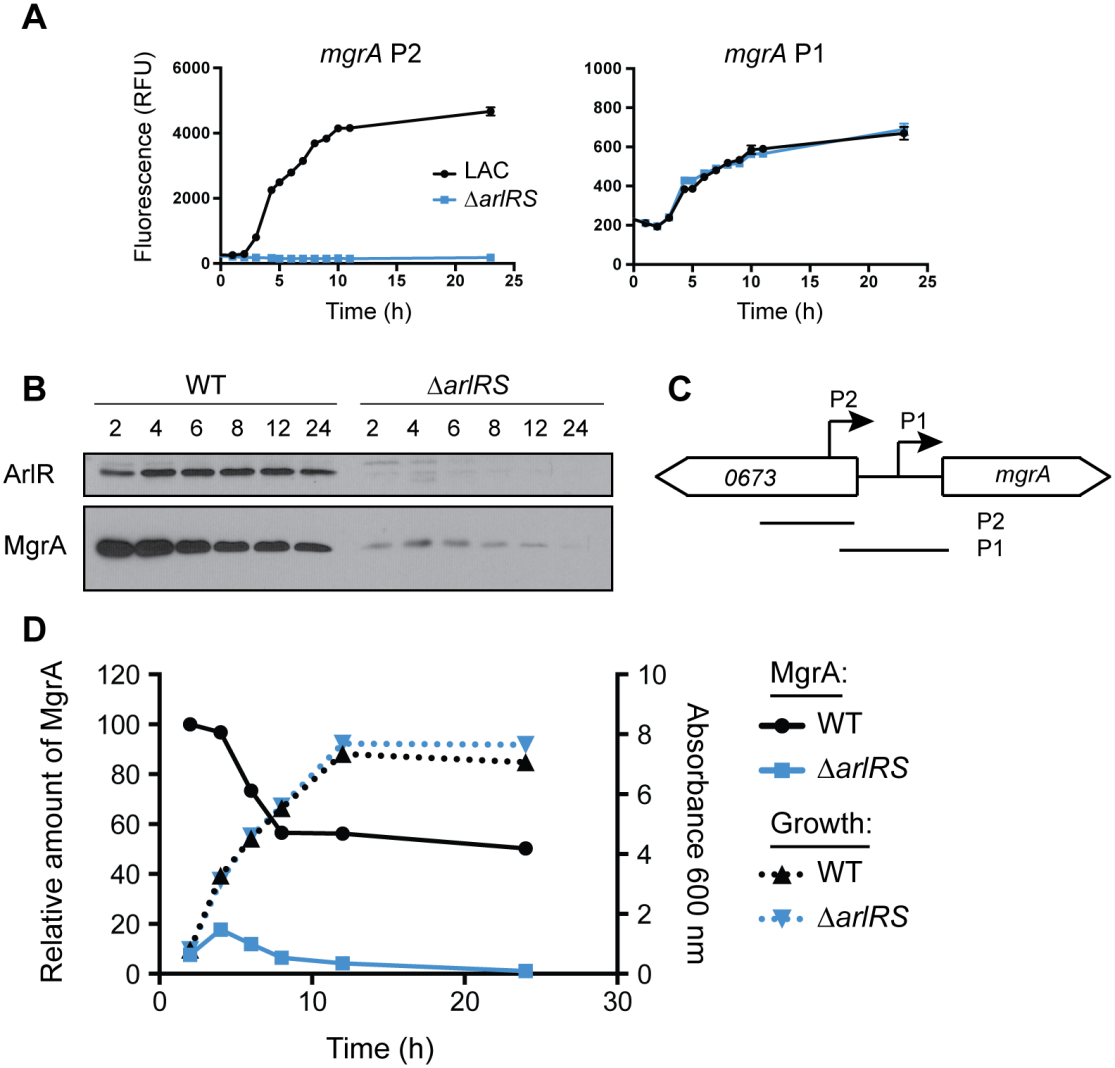
ArlRS is required for *mgrA* expression. The *mgrA* gene has two promoters, depicted in (C). Lines labeled P2 and P1 show the fragments used to make transcriptional reporters. (A) Each *mgrA* promoter was independently cloned upstream of a promoterless copy of GFP in plasmid pCM11. Expression from the upstream promoter *mgrA* P2 (left) and downstream *mgrA* P1 (right) was assessed in the WT strain LAC (black) and the *arlRS* mutant (blue). (B) Western blot showing ArlR and MgrA protein levels at various time points (hours). (D) Quantification of MgrA protein levels in LAC (black, solid line) and the *arlRS* mutant (blue, solid line), and growth curves of the same strains (dotted lines). Quantification is representative of three separate experiments.

To confirm that ArlRS regulates MgrA, we used a combination of transcriptional reporters and Western blots. The *mgrA* gene has two promoters located 302 nucleotides (P2) and 124 nucleotides (P1) upstream of the start codon [38]. We amplified each of these promoters and fused them to GFP to generate transcriptional reporters (Fig 4C). Expression from *mgrA* P2 was entirely dependent on ArlRS, whereas expression from *mgrA* P1 was unchanged in the *arlRS* mutant compared with LAC (Fig 4A). In addition, under the conditions of this assay it appears that *mgrA* P2 may be ~10-fold stronger than *mgrA* P1. Detection of MgrA protein levels by Western blot confirmed that there was ~80–95% less MgrA produced in the *arlRS* mutant throughout the growth curve (Fig 4B and 4D). This sharp decrease in MgrA protein levels in the *arlRS* mutant likely explains why *arlRS* and *mgrA* mutants have similar phenotypes, despite the observation that ArlRS only regulates one of the *mgrA* promoters.

### MgrA represses *ebh*

**Fig 5:**
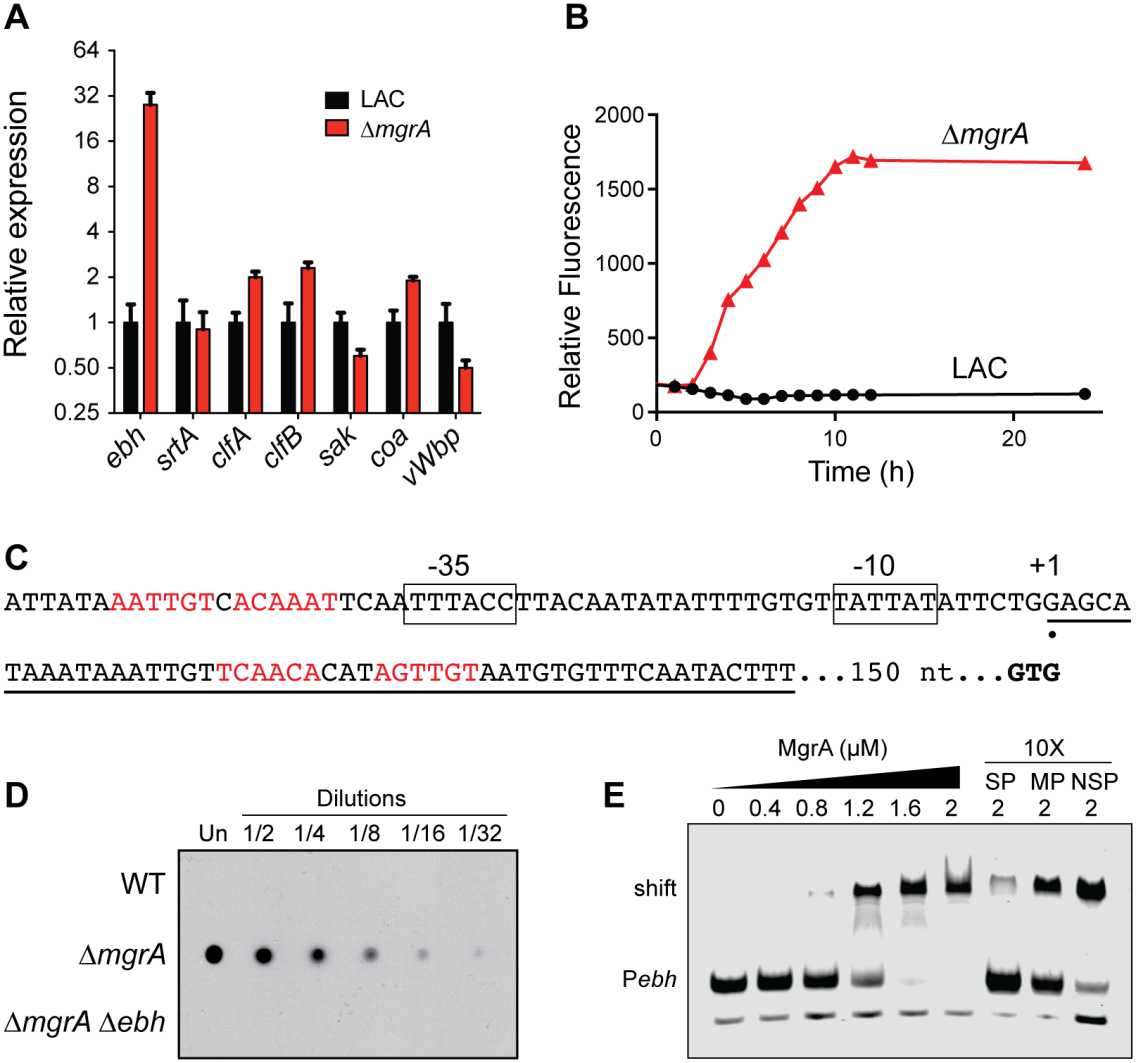
MgrA directly represses *ebh*. (A) Quantification of transcripts of genes related to clumping in LAC and the *mgrA* mutant using qRT-PCR. Values are averages and standard deviations of three biological replicates, normalized to expression in LAC for each gene. (B) Expression of *ebh* was measured in LAC and the *mgrA* mutant using a transcriptional fusion of the entire intergenic region upstream of *ebh* to GFP. (C) The putative *ebh* transcription start site, indicated by the black circle, was determined by 5’ RACE. Possible -10 and -35 promoter elements are boxed, and the *ebh* start codon (GTG) is shown in bold. Potential MgrA binding sites are shown in red text, and the sequence of the DNA probe used for EMSA experiments is underlined. (D) Ebh protein levels were measured by dot blot in 2-fold dilutions of supernatants from overnight cultures of the indicated strains. All strains also lacked the *spa* gene. (E) EMSA showing MgrA binding to the *ebh* promoter. Increasing concentrations of MgrA were incubated with an IRDye-labeled probe (P_*ebh*,_ sequence underlined in C) before separation by PAGE. Unbound probe (P_*ebh*_) and MgrA-probe complex (shift) are indicated. The last three lanes show competition experiments, where the binding reaction included a 10-fold excess of unlabeled specific probe (SP), non-specific probe (NSP), or a version of the specific probe in which the proposed MgrA binding site was mutated (MP).

To understand how MgrA controls clumping, we used qRT-PCR to investigate if MgrA regulates expression of genes known to affect clumping and coagulation. Although several cell wall associated proteins in *S*. *aureus* have been reported to interact with fibrinogen [6], under the conditions of these in vitro experiments it appears that ClfA is the dominant adhesin required for clumping with both plasma and fibrinogen ([33], S1 Fig). Genes for the clumping factors *clfA* and *clfB* showed modest increases in expression in the *mgrA* mutant (Fig 5A), which would be expected to enhance rather than inhibit clumping. Notably, expression of *ebh* increased 28-fold in the *mgrA* mutant, whereas changes in all other genes tested were <2.5-fold. There was very little change in expression of the coagulase genes *coa* and *vWbp*, the plasminogen activator staphylokinase (*sak*), or in the sortase gene *srtA*. Thus, of the genes tested, it seems that MgrA is most likely to affect clumping through repression of *ebh*.

We confirmed that MgrA regulates *ebh* by measuring expression of an *ebh* transcriptional reporter in which the *ebh* promoter region was fused to GFP. As seen previously [33], expression of *ebh* was very low in the wild type strain when growing in rich media (Fig 5B). In contrast, expression of the P_*ebh*_-GFP fusion was much higher in the *mgrA* mutant, suggesting that MgrA represses *ebh*. Likewise, detection of Ebh protein levels by dot blot showed a substantial increase in the *mgrA* mutant (Fig 5D) that was specific for Ebh, as there was no signal in the *mgrA ebh* double mutant.

To investigate MgrA binding in greater detail, we mapped the *ebh* promoter using 5’ RACE (Fig 5C). We identified one putative transcription start site located 200 nucleotides upstream of the GTG start codon. Putative -10 and -35 promoter elements are shown in Fig 5C. MgrA was previously shown to bind to the six-nucleotide sequence (A/T)GTTGT [41]. As a member of the MarR/SlyA family of dimeric DNA binding proteins [42], MgrA likely binds to closely spaced inverted repeats of this hexameric sequence. There are at least two potential MgrA binding sites in the vicinity of the *ebh* promoter, shown in red in Fig 5C. One site, centered 24 nucleotides downstream of the putative transcription start site, matches the consensus sequence perfectly. A second potential MgrA binding site is centered 11 nucleotides upstream of the putative -35 element. We tested if MgrA is able to interact directly with the *ebh* promoter using a 50 bp DNA probe spanning the downstream potential MgrA binding site (Fig 5E). We found that MgrA was able to bind to the *ebh* promoter probe, and this binding could be outcompeted with a 10-fold excess of an identical unlabeled probe. A version of this competitor probe with a mutated potential MgrA binding site was poor competitor, however, and a 10-fold excess of a non-specific probe was not able to compete for MgrA binding. Taken together, these results indicate that MgrA represses *ebh* by directly interacting with the *ebh* promoter.

**Fig 6:**
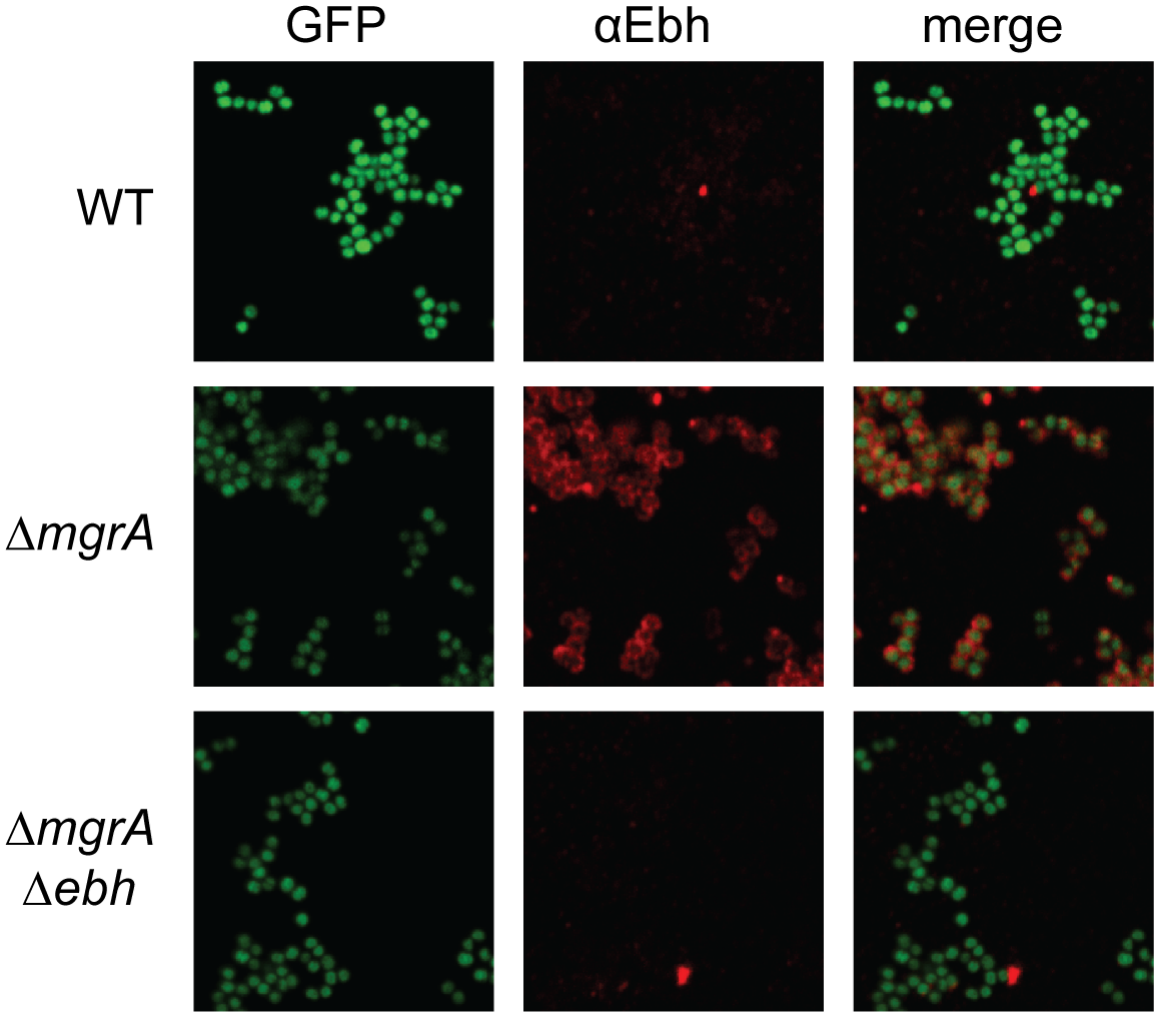
Immunofluorescence microscopy showing overproduction of Ebh in the *mgrA* mutant. All strains constitutively expressed GFP and were probed for Ebh using Alexa fluor 568-labeled secondary antibodies, as described in the methods.

Lastly, we used immunofluorescence microscopy to visualize Ebh presence and localization in the wild type and *mgrA* mutant (Fig 6). All strains also lacked *spa*, the gene encoding Protein A, to avoid potential non-specific antibody binding. There was essentially no Ebh visible in the wild type strain or in the *mgrA ebh* double mutant. The *mgrA* mutant, however, showed abundant Ebh localized to the cell surface (Fig 6), consistent with our observations that *ebh* is up-regulated in an *mgrA* mutant.

### Comparison of clumping in a variety of *S*. *aureus* strains

**Fig 7:**
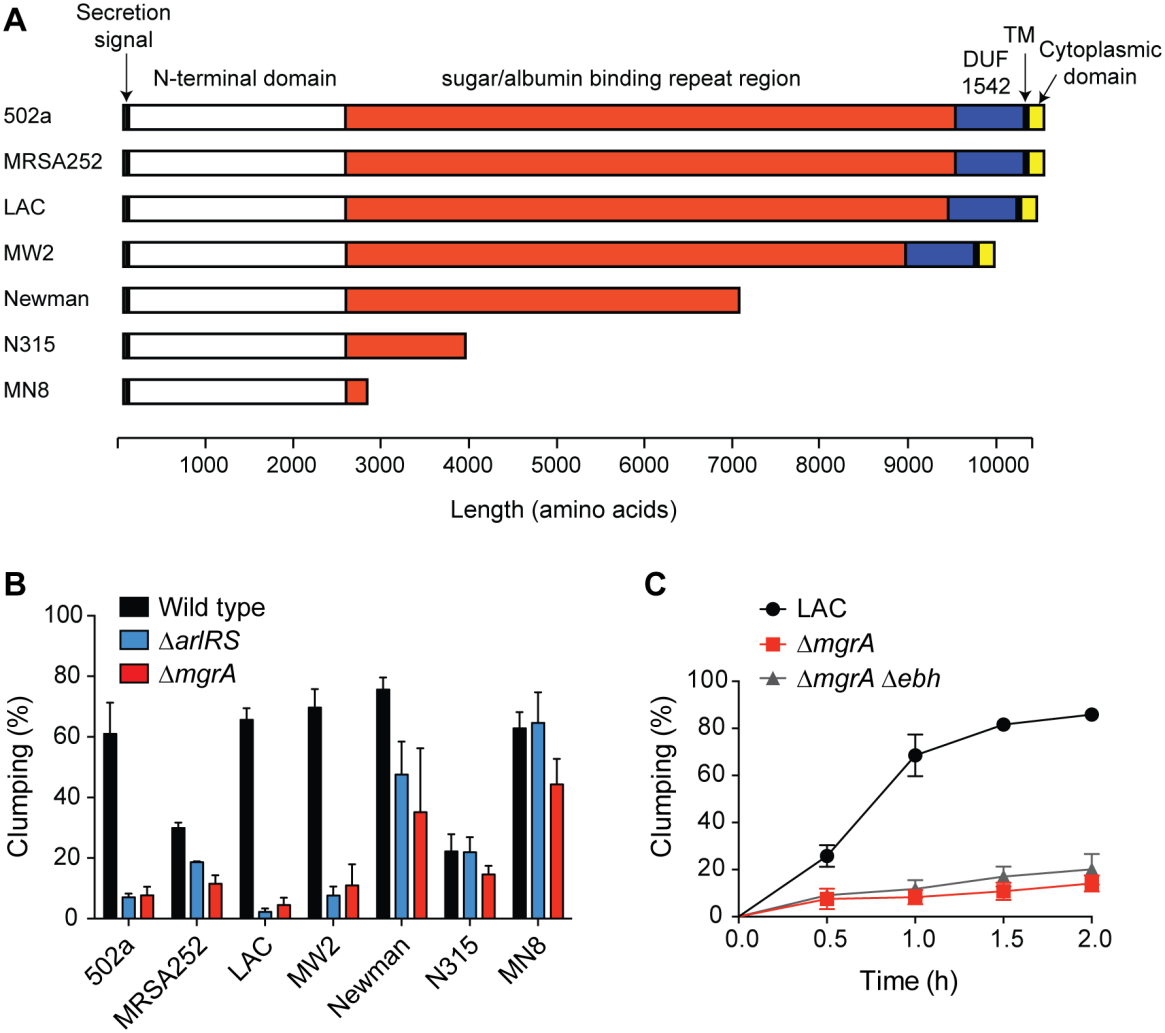
Comparison of clumping phenotypes in strains with truncated *ebh* genes. (A) Schematic of predicted Ebh proteins from a collection of *S*. *aureus* strains, showing only the product of the first predicted *ebh* ORF for each strain. Key domains, as well as the secretion signal sequence and transmembrane (TM) domain are indicated. (B) Clumping of *arlRS* and *mgrA* mutants in each of the strains shown in (A) after 1 h of incubation with human plasma. (C) Clumping time course of LAC, *mgrA* mutant and *mgrA ebh* double mutants incubated with human plasma. All clumping results represent averages of three separate experiments.

Many, but not all, strains of *S*. *aureus* encode a full-length copy of *ebh*. Truncated versions of *ebh* lack the trans-membrane domain, meaning the resulting protein should no longer be membrane anchored (Fig 7A). Because of this strain variability, we were interested in comparing behavior of *mgrA* and *arlRS* mutants across of range of strains. We hypothesized that truncations in Ebh might mask *mgrA* and *arlRS* mutant clumping phenotypes. Sequenced strains with full-length copies of *ebh* included 502a, the USA200 strain MRSA252, and the USA400 strain MW2. Strains with truncations in *ebh* included Newman, the USA100 strain N315, and the USA200 strain MN8. Most strains clumped with similar kinetics to LAC, although MRSA252 and N315 were noticeably slower, which could be due to a number of factors, potentially including decreased expression of clumping factors. In general, strains with full-length copies of *ebh* had a large clumping defect when either *mgrA* or *arlRS* was inactivated (Fig 7B). Newman had an intermediate phenotype, similar as previously noted [33], consistent with it expressing a version of Ebh that is nearly full-length but not membrane-anchored. Lastly, N315 and MN8, which encoded the shortest variants of *ebh* that we tested, had essentially no clumping defect when *mgrA* or *arlRS* were deleted (Fig 7B). These results suggest that there is a correlation between expression of an intact version of Ebh and inhibition of clumping. In light of this observation, we constructed an LAC strain lacking both *mgrA* and *ebh*. Surprisingly, however, deleting *ebh* did not restore clumping to an *mgrA* mutant, suggesting that additional factors were involved (Fig 7C).

### Identification of genes regulated by MgrA

We hypothesized that the *mgrA* mutant was overproducing multiple surface proteins, including Ebh, that were interfering with clumping. Luong *et al*. [37] used microarray analysis to identify 355 genes that were regulated by MgrA in Newman. These included genes for the surface proteins FmtB and Protein A (Spa), in addition to Ebh. Initial tests with triple mutants lacking *mgrA*, *ebh*, and either *spa* or *fmtB* did not restore clumping (discussed in more detail below). Thus we decided to identify genes regulated by *mgrA* in LAC using RNA sequencing (RNA-seq), speculating that there may be variation between genes regulated by MgrA in Newman and LAC.

LAC and the isogeneic *mgrA* mutant were grown in rich medium to an optical density of 1.5, a time point at which MgrA is highly expressed (Fig 4D). We identified 104 genes whose expression was ≥4-fold different at 95% confidence, listed in S1 Table. There were 55 genes with increased expression in the *mgrA* mutant and 49 genes with decreased expression, including *mgrA* itself. In agreement with previous studies [37,38,43], expression of the capsule genes *cap5A*, *cap5B*, *cap5C*, and *cap5E* was modestly decreased in the *mgrA* mutant. In addition, *ebh* was upregulated ~45-fold in the *mgrA* mutant, consistent with our qRT-PCR measurements (Fig 5A). Whether MgrA directly or indirectly regulates expression of these genes remains to be determined. While the half-site consensus binding sequence for MgrA is thought to be (A/T)GTTGT [41], the preferred spacing between half-sites and the tolerance for variability remain unknown, making it difficult to distinguish MgrA binding sites without experimental validation. Some of the genes identified by RNA-seq are likely to be indirectly regulated by MgrA, though, because MgrA represses expression of at least two transcriptional regulators, SarV and AtlR (S1 Table).

The genes for eight known and putative surface proteins were significantly upregulated in the *mgrA* mutant, including *ebh* (Table 1). All except Ebh contain an LPXTG motif and are predicted to be cell wall anchored. Several are fairly well studied, including protein A (Spa), which binds IgG, and the fibronectin-binding protein FnbB. SdrD, a member of the serine-aspartate repeat family, and SasG are both reported to contribute to adherence to desquamated nasal epithelial cells [44,45]. There is limited information about FmtB and SasC, which both contain multiple repeats of the domain of unknown function DUF1542 (a domain also present in Ebh), although SasC has been shown to promote biofilm formation [46]. Finally, SraP is a member of the serine-rich repeat (SRR) glycoprotein family involved in adhesion to platelets, likely through binding to sialylated glycoproteins [47–49].

**Table 1:** Surface proteins regulated by MgrA.

Gene	Fold Change in Δ*mgrA* vs LAC	Function
*ebh*	+45	extracellular matrix binding protein homolog
*fmtB*	+41	truncated FmtB protein
*spa*	+22	immunoglobulin G binding protein A
*sdrD*	+20	serine-aspartate repeat-containing protein D
*sasG*	+8.3	truncated surface protein G
*sasC*	+7.7	surface protein C
*fnbB*	+5.4	fibronectin binding protein B
*sraP*	+5.0	serine rich adhesin for platelets

**Fig 8:**
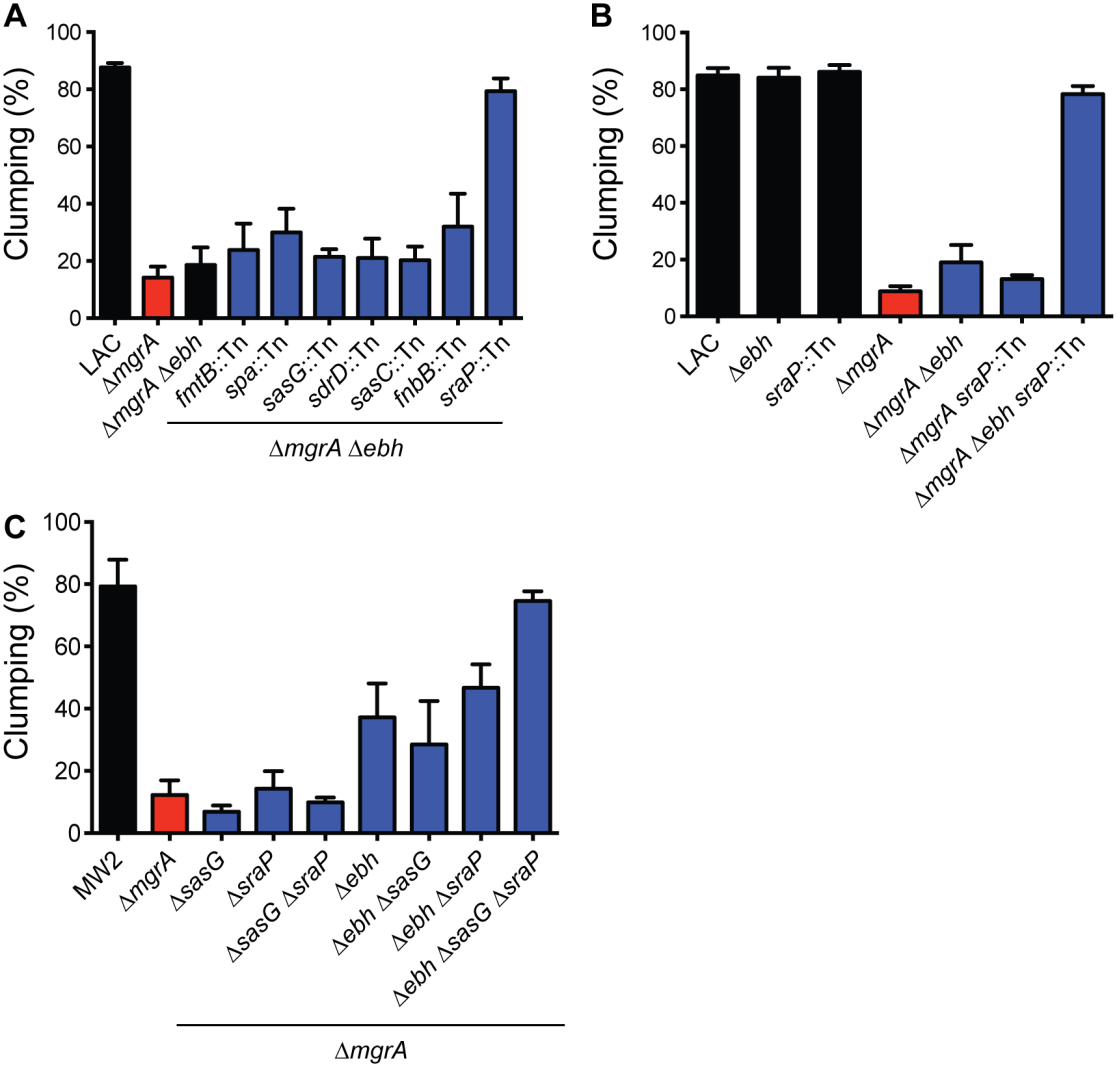
Identification of other surface proteins involved in inhibiting clumping. (A) Genes encoding surface proteins predicted to be regulated by MgrA were inactivated in an *mgrA ebh* double mutant background, and clumping was measured after 2 h of incubation with human plasma. (B) Comparison of clumping in LAC strains lacking combinations of *mgrA*, *ebh*, and *sraP*, measured after incubation with human plasma for two hours. (C) Clumping of MW2 strains lacking combinations of *mgrA*, *ebh*, *sraP*, and *sasG*. Graph shows clumping after 1 h of incubation with human plasma. All clumping results represent averages of three separate experiments.

We mutated each of these genes individually in the Δ*mgrA* Δ*ebh* background using transposon insertions from the Nebraska Transposon Mutant Library (NTML) [50] and tested for restoration of clumping (Fig 8A). The *mgrA ebh sraP* triple mutant was able to clump almost as well as the LAC wild type strain, suggesting that SraP is involved in blocking clumping. Comparison of *mgrA ebh* and *mgrA sraP* double mutants showed that only the *mgrA ebh sraP* triple mutant was able to restore clumping (Fig 8B). This suggests that Ebh and SraP are redundant in this situation, and up-regulation of either SraP or Ebh is sufficient to block clumping in LAC. Therefore genes for both surface proteins must be disrupted to restore clumping to an *mgrA* mutant. However, in MW2 it was not sufficient to delete *ebh* and *sraP* to restore clumping in the *mgrA* mutant (Fig 8C). Unlike LAC, MW2 encodes a full-length copy of SasG, which is also predicted to be regulated by MgrA (Table 1). We hypothesized that up-regulation of SasG in the MW2 *mgrA* mutant may also be contributing to inhibition of clumping, and indeed an *mgrA ebh sraP sasG* quadruple mutant was able to clump as well as the wild type parent strain (Fig 8C). These results suggest that *mgrA* mutants are unable to clump due to up-regulation of surface proteins such as Ebh, SraP, and SasG that interfere with clumping.

### *sraP* is regulated by MgrA

**Fig 9:**
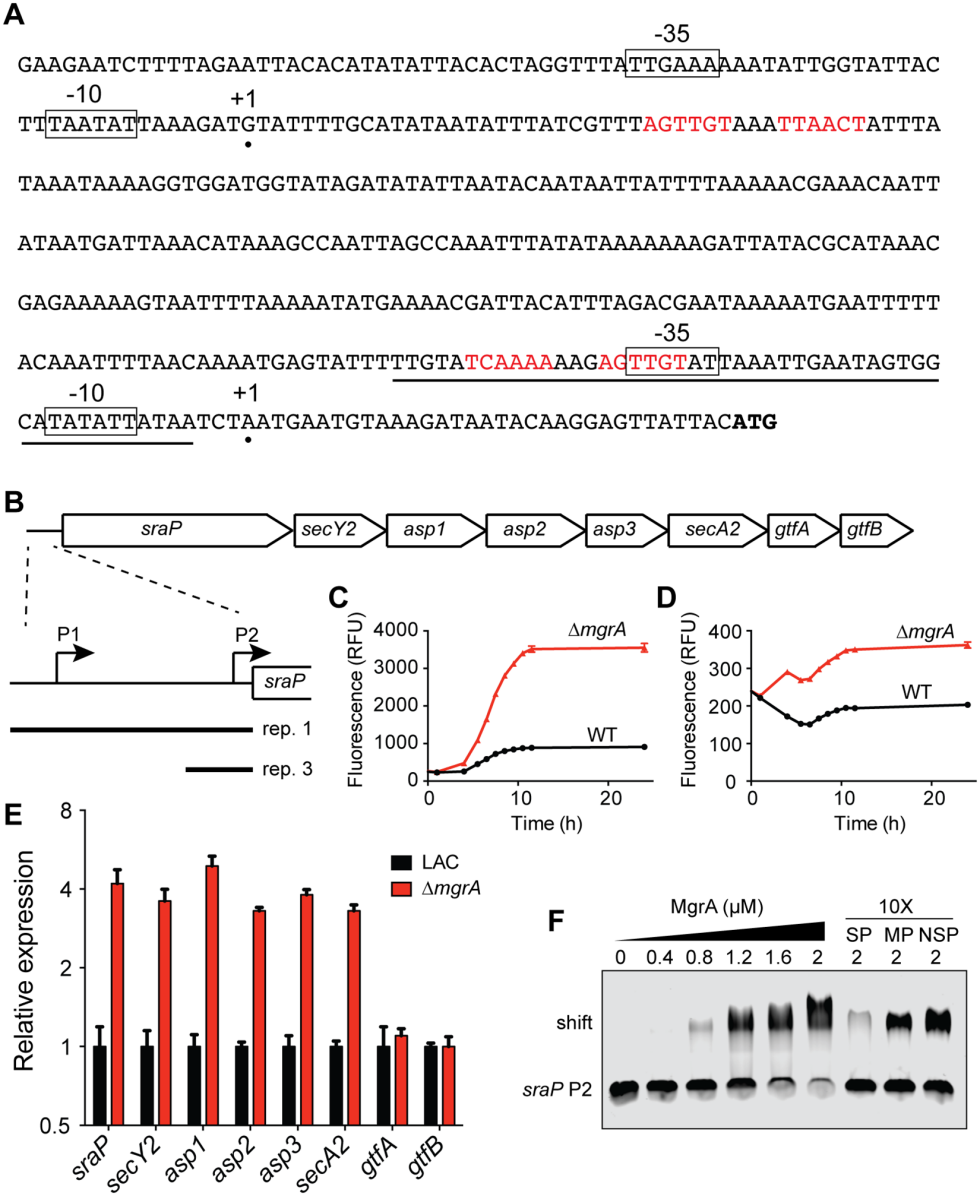
MgrA directly represses *sraP*. (A) The *sraP* promoter region, showing the two putative transcription start sites (marked with black circles) determined using 5’ RACE. Potential -10 and -35 promoter elements are boxed, and possible MgrA binding sites are shown in red text. The *sraP* ATG start codon is indicated in bold font. The promoter probe used for EMSA experiments is underlined. (B) Schematic of the *sraP* gene cluster, including its dedicated secretory system (*secY2*, *asp1-3*, and *secA2*) as well as the putative glycosyltransferases *gtfA* and *gtfB*. Inset below shows the two promoters mapped in (A) and the regions used to make transcriptional reporter plasmids (labeled rep 1 and rep 3). (C) Expression of a GFP transcriptional fusion containing both P1 and P2 (labeled rep 1 in panel B) in LAC and the isogenic *mgrA* mutant. (D) Expression of transcriptional reporter 3, containing just *sraP* P2 driving production of GFP. (E) Expression of each gene in the *sraP* gene cluster in LAC and the *mgrA* mutant, measured by qRT-PCR. Values are averages and standard deviations of three biological replicates and are normalized to expression in LAC for each gene. (F) EMSA assessing MgrA binding to the *sraP* P2 promoter. Increasing concentrations of purified MgrA were incubated with the probe underlined in panel A. Unbound probe and shifted probe are indicated on the left. In the last three lanes competition with a 10-fold excess of unlabeled probes was assessed. SP, specific probe identical to the labeled probe; MP, specific probe in which the putative MgrA binding site has been mutated; NSP, non-specific probe.

SraP is unusual in that it is glycosylated and exported by its own secretion system, consisting of the secretory proteins SecY2 and SecA2, three accessory secretory proteins (Asp1-3), and two putative glycosyltransferases, GtfA and GtfB [47,51]. All eight genes are co-localized on the chromosome (Fig 9B), but little is known about their expression. Our RNA-seq data indicated that the first five genes in the cluster (*sraP*, *secY2*, *asp1*, *asp2*, and *asp3*) were all up-regulated between 4.7 and 5.5-fold in the *mgrA* mutant (S1 Table). qRT-PCR analysis confirmed that expression of these five genes, as well as *secA2*, was increased ~4-fold in the *mgrA* mutant, whereas there was essentially no change in expression of the last two genes, *gtfA*, and *gtfB* (Fig 9E). Thus, MgrA appears to regulate expression of *sraP* and its secretion apparatus, but not the two downstream glycosyltransferases that are believed to modify SraP.

We used 5’ RACE to map the *sraP* transcription start site(s) and identified two putative promoters (Fig 9A and 9B). The first promoter, P1, initiates transcription 343 nucleotides upstream of the *sraP* start codon, and the second promoter, P2, is located 33 nucleotides upstream of the start codon. Interestingly, there are potential MgrA binding sites overlapping the putative -35 sequence of P2 and centered 35 bp downstream of the P1 transcription start site (shown in red in Fig 9A). To test if MgrA regulates both *sraP* promoters, we constructed two transcriptional fusions to GFP. The first fusion included both P1 and P2 (Fig 9C), whereas the second fusion contained only P2 (Fig 9D). Both fusions showed increased expression in the *mgrA* mutant, although overall expression from the fusion containing P1 and P2 was ~10-fold higher. These results suggest that P1 is the major promoter under these conditions, and that both P1 and P2 are controlled by MgrA.

We performed EMSAs with purified MgrA to see if it could bind to the putative MgrA binding site overlapping P2 (Fig 9F). MgrA was able to bind in a dose-dependent manner, producing a distinct shifted band. This binding could be outcompeted by adding a 10-fold excess of unlabeled probe, but not by adding the same unlabeled probe in which the putative MgrA binding site had been mutated (Fig 9F). Likewise, addition of a 10-fold excess of a non-specific probe from within the *sraP* coding sequence did not affect MgrA binding.

### MgrA is required for endocarditis in MW2 and 502a

We have previously shown that an *arlRS* mutant is less virulent in a rabbit model of endocarditis [33]. Compared to the wild type strain MW2, the *arlRS* mutant was less lethal, formed smaller heart valve vegetations, and had lower bacterial burdens within the vegetations [33]. We hypothesized that this was due in part to the *arlRS* mutant’s defects in clumping and fibrinogen binding, and indeed, virulence could be partially restored by also deleting *ebh*. Since MgrA is also required for clumping, we predicted that an *mgrA* mutant would be less able to cause endocarditis. *S*. *aureus* strain Newman *mgrA* mutants have been shown to be less virulent in mouse sepsis, septic arthritis, and abscess models [42,52,53]. However, the requirement for MgrA to cause endocarditis is not clear, as a rat endocarditis model of a Newman *mgrA* mutant had lower bacterial burdens within vegetations [54], but in a rabbit model COL and MW2 *mgrA* mutants behaved like the wild type strains [55].

To revisit if MgrA is required for endocarditis, we constructed *mgrA* deletions in MW2 and the recently sequenced strain 502a [56]. 502a was used in the 1960s to deliberately colonize newborns, which provided some protection from other virulent *S*. *aureus* strains, but was halted after a child died of pneumonia caused by 502a [57]. These strains were chosen because *mgrA* mutants in both backgrounds had clumping defects similar to that seen with LAC (Fig 7B). Unlike LAC and other USA300 isolates, both MW2 and 502a cause endocarditis in this model without immediately killing the rabbits.

**Fig 10:**
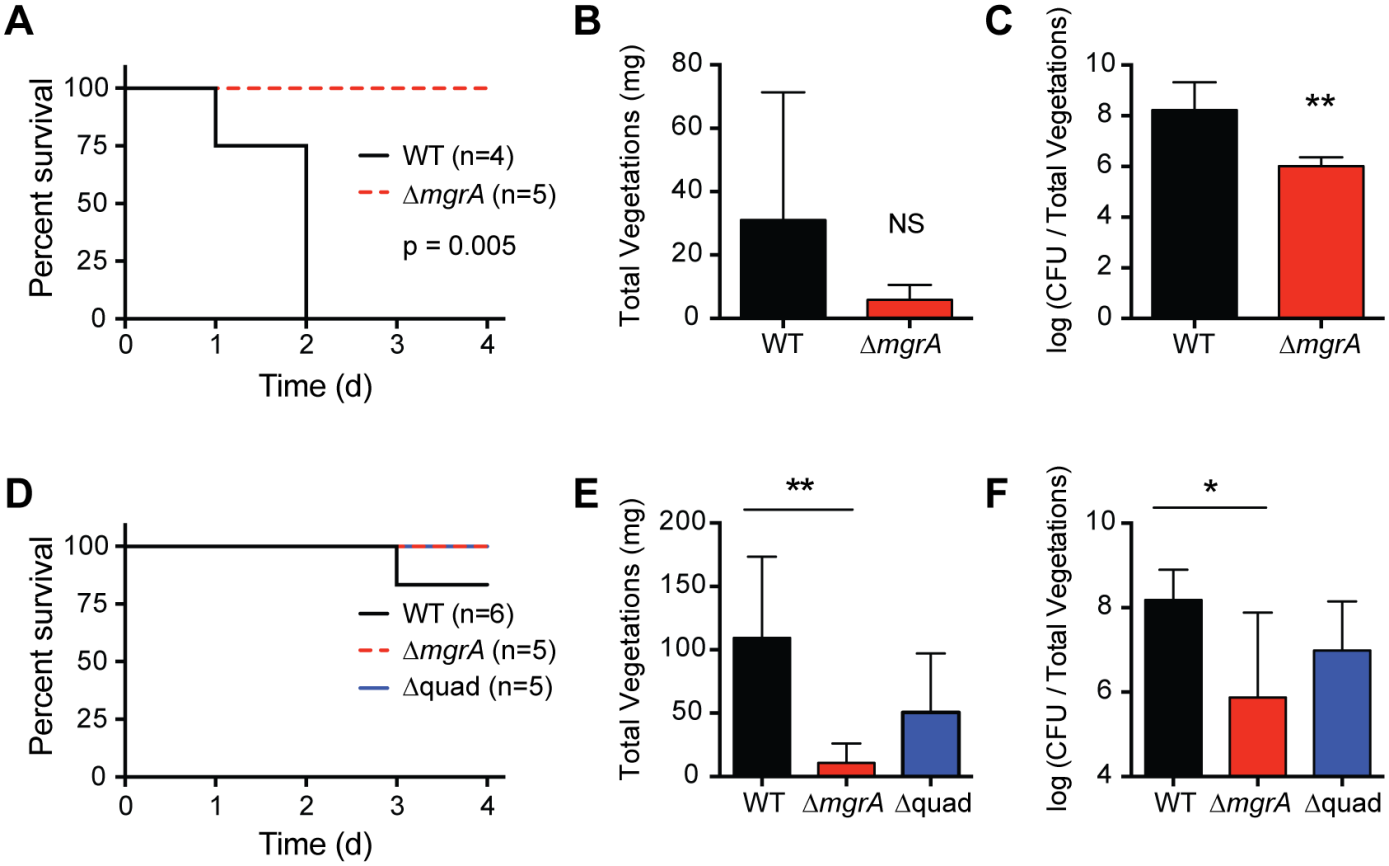
*mgrA* mutants are less virulent in a combined rabbit model of sepsis and endocarditis. Prior to infection, aortic valve damage was induced by temporary placement of plastic catheters in the carotid artery. Catheters were removed and rabbits were infected intravenously with either strain 502a (A-C), or strain MW2 (D-F). WT strains were compared to the *mgrA* single mutant, and for MW2 the *mgrA ebh sraP sasG* quadruple mutant (Δquad) was also tested. Rabbits were monitored for up to four days, and survival (A, D), total heart valve vegetation weights (B, E), and CFUs per total vegetations (C, F) were monitored. Statistical significance was determined using a log-rank (Mantel-Cox) test (survival), or two-tailed t test (vegetation weights and CFU counts). ** p<0.005, * p<0.05, NS not significant.

In studies of sepsis and infective endocarditis, we damage the aortic valves for 2 hours, then remove the catheters, followed by intravenous administration of microbes by the marginal ear vein. This procedure allows us to study infection development more resembling human disease with the absence of biofilm formation on catheters, in contrast to models that leave the catheters in place [54,55]. In our model, the wild type 502a was significantly more lethal than the *mgrA* knockout, with all 4 rabbits receiving 502a succumbing by day 2 post infection (Fig 10A). In contrast, all 5 animals receiving the same dose of 502a *mgrA* mutant survived the entire 4 day test period (Fig 10A). Total vegetation weights in the wild type-treated animals was greater than in animals given the *mgrA* mutant (Fig 10B); however the differences were not statistically significant, likely because vegetation sizes were being compared between animals that succumbed by day 2 compared to not dying. Total vegetation CFUs were significantly higher in 502a-treated animals (Fig 10C) than in rabbits given the *mgrA* mutant.

When rabbits were infected with strain MW2, one of the animals treated with wild type succumbed over the 4 day period, whereas none of the animals infected with the *mgrA* mutant died (Fig 10D). Both total vegetation weights (Fig 10E) and total CFUs (Fig 10F) were significantly higher in animals treated with MW2 than in animals treated with the MW2 *mgrA* mutant. We also tested if virulence was restored in the MW2 *mgrA ebh sraP sasG* quadruple mutant (Δquad). All rabbits infected with the quadruple mutant survived the four day time course, similar to the wild type and *mgrA* single mutant (Fig 10D). Total vegetation weights were higher than in the animals treated with the *mgrA* mutant (Fig 10E), although this difference was not statistically significant. Likewise, the total CFUs were higher in rabbits infected with the quadruple mutant compared to treatment with the *mgrA* mutant (Fig 10F). These results suggest that overexpression of surface proteins contributes to the lack of fitness of the *mgrA* mutant *in vivo*, although other factors likely contribute to its decreased virulence.

### MgrA controls biofilm formation through regulation of surface proteins

**Fig 11:**
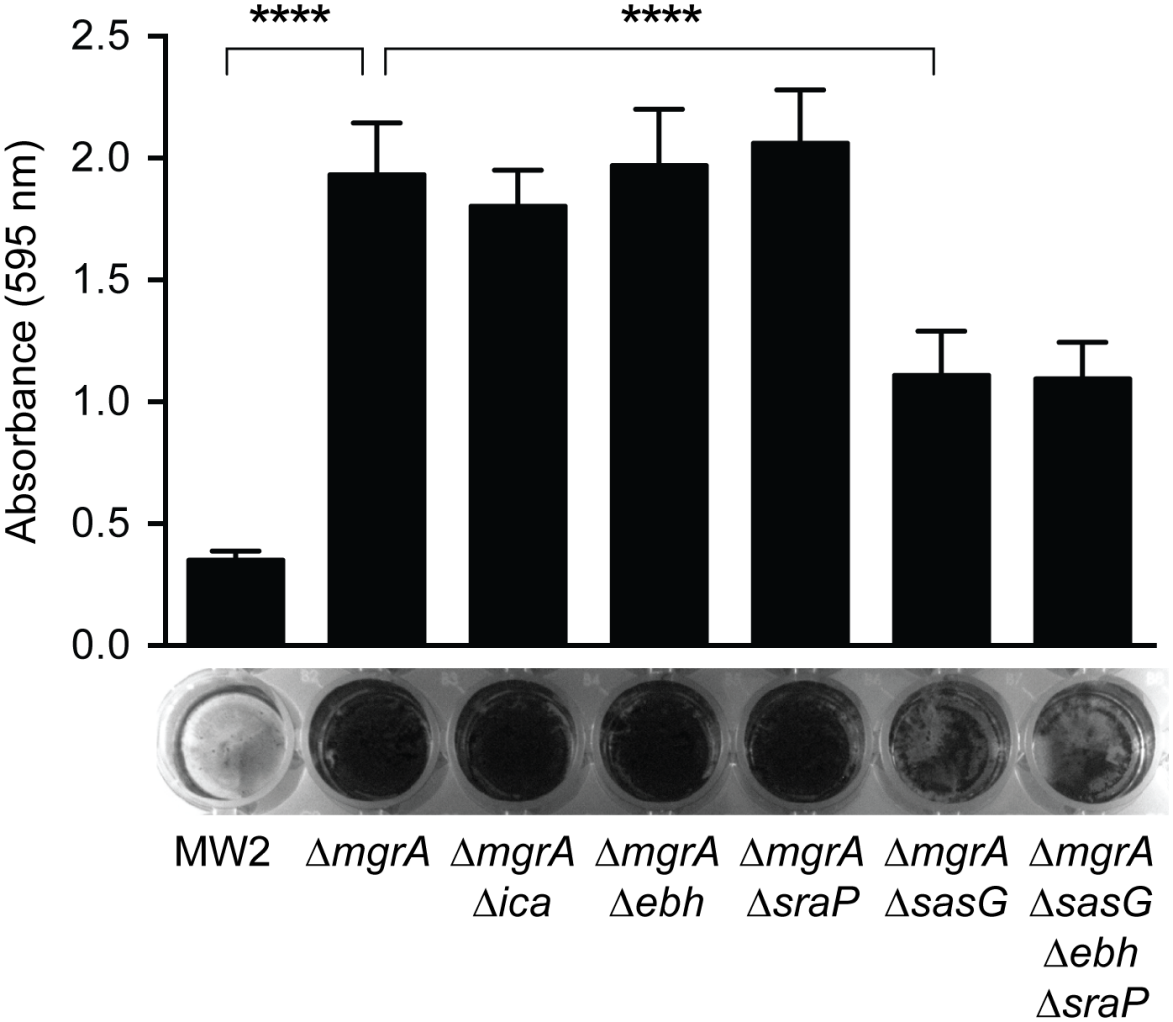
Increased biofilm formation in an *mgrA* mutant is partially due to up-regulation of SasG. Cultures were incubated statically for 16 h in BHI + 0.25% glucose, and biofilm biomass was assessed by staining with crystal violet. Solubilized crystal violet was quantified by measuring absorbance at 595 nm. Values represent averages and standard deviations of two separate experiments with six replicate wells each. Statistical significance was determined using a two-tailed t test; **** p<0.0001.

Differential expression of surface proteins is also likely to affect biofilm formation by *S*. *aureus*. In contrast to our observations that *mgrA* mutants have a clumping defect, strains lacking *mgrA* show increased biofilm formation [39,40,58]. This increase in biofilm formation appears to be particularly pronounced in strains that produce SasG, such as MW2 and SH1000 [39]. SasG is known to promote biofilm formation by facilitating intercellular adhesion [59,60], and we predicted that increased production of SasG in *mgrA* mutants could explain the observed enhancement in biofilm formation. In agreement with previous results, we observed increased biofilm formation in the MW2 *mgrA* mutant (Fig 11). To test if this enhanced biofilm formation was due to increased production of extracellular polysaccharide PIA (polysaccharide intracellular adhesin), we constructed a double mutant lacking both *mgrA* and the *icaADBC* locus responsible for production of PIA. The *mgrA ica* mutant produced as much biofilm as the *mgrA* single mutant, indicating that the increase in biofilm formation is PIA-independent. However, the *mgrA sasG* mutant formed significantly less biofilm than the *mgrA* single mutant. Deletion of *ebh* or *sraP* had no effect, suggesting that the enhanced biofilm formation in the MW2 *mgrA* mutant is primarily due to increased expression of SasG.

## Discussion

**Fig 12:**
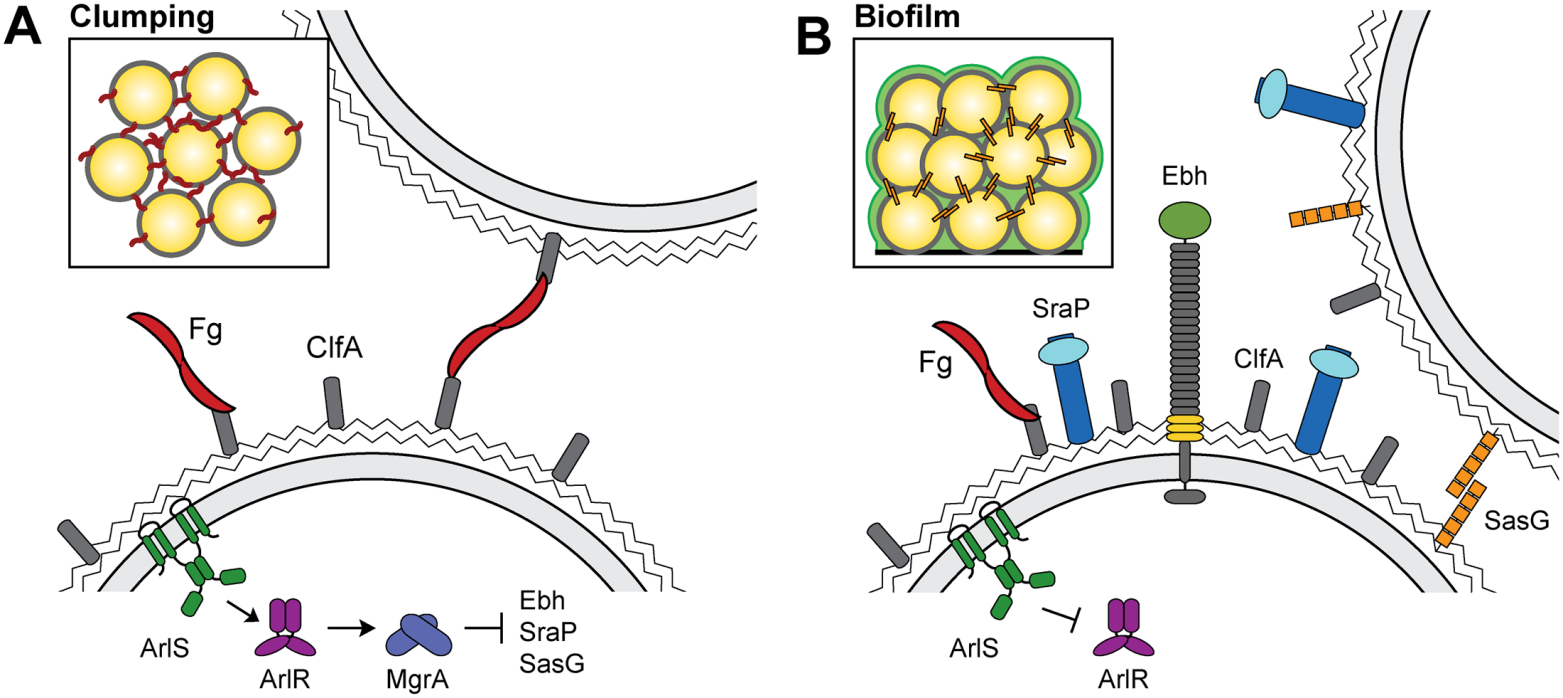
Proposed model for how the ArlRS-MgrA regulatory cascade controls clumping (A) and biofilm formation (B). In response to an unknown signal, the ArlRS two component system activates expression of MgrA, which in turn represses expression of the large surface proteins Ebh, SraP, and SasG. ClfA interacts with fibrinogen, allowing clumping to occur (inset). When ArlRS is inhibited or inactivated, MgrA production is diminished and expression of Ebh, SraP, and SasG is de-repressed (B). These surface proteins interfere with clumping, while up-regulation of SasG can also promote biofilm formation (inset). Fg, fibrinogen.

The ArlRS TCS has been linked to virulence phenotypes multiple times [31–33], but we are only beginning to understand how this regulatory system functions. We demonstrate here that ArlR activates expression of the global regulator MgrA, and that *mgrA* mutants, like *arlRS* mutants, show a pronounced defect in clumping in the presence of fibrinogen. Numerous studies, including this one, have shown that MgrA is important for virulence [42,52–54,58], although the reason for this has been largely unclear. Our RNA-seq findings indicate MgrA affects the expression of >100 genes, including eight surface proteins that are likely to be important for adhesion and immune evasion within the host. These results support the idea that ArlRS and MgrA constitute a regulatory cascade that, in response to an unknown signal, profoundly changes expression of cell wall associated proteins, perhaps allowing *S*. *aureus* to adapt to a new niche within the host or progress to a different disease stage. A proposed model for how the ArlRS-MgrA cascade affects clumping and biofilm formation is shown in Fig 12.

Both *arlRS* and *mgrA* mutants in a variety of strain backgrounds fail to clump in the presence of fibrinogen or plasma. We show that this is due to increased expression of surface proteins such as Ebh, SraP, and SasG, which may interfere with clumping by steric hindrance. The ability of *S*. *aureus* to interact with fibrinogen and form clumps appears to be important for establishing infections such as septicemia and infective endocarditis [22,23,25,26]. Using a rabbit model of infective endocarditis, we show that *mgrA* mutants form smaller vegetations, and that the bacterial burden within these vegetations is lower. This virulence defect can be partially restored by deleting the genes for Ebh, SraP, and SasG, suggesting that up-regulation of surface proteins contributes to the virulence defect of the *mgrA* mutant. We predict that clumping may also be important for abscess formation. There is growing evidence that staphylococcal abscesses consist of a dense core of bacterial cells, called the staphylococcal abscess community, surrounded by a fibrin “pseudocapsule” [61–63]. Formation of this fibrin layer is dependent on the action of the staphylococcal coagulases and likely protects the bacteria from clearance by the immune system [61,63]. These abscess communities are likely to be held together by fibrin/fibrinogen, similar to the dense clumps generated *in vitro* with purified fibrinogen. Indeed, *mgrA* mutants are attenuated in a kidney and liver abscess model [42].

Among the genes repressed by MgrA are those encoding eight surface proteins: Ebh, FmtB, Spa (protein A), SdrD, SasG, SasC, FnbB, and SraP. By size, Ebh and SraP are two of the largest extracellular proteins made by *S*. *aureus*, at 1.1 MDa for Ebh, and 228 kDa for SraP before glycosylation. We propose that increased production of Ebh and SraP at the cell surface physically interferes with the fibrinogen cross-linking between cells that is required for clumping. Unlike LAC, strain MW2 encodes a full-length copy of SasG that also appears to contribute to blocking clumping. A similar phenomenon was observed previously when SasG was overproduced from a plasmid [45,59]. SasG is normally undetectable under *in vitro* growth conditions, but when it was artificially produced at high levels it was able to interfere with adhesion of cells to fibrinogen and fibronectin, without affecting expression of other surface proteins like ClfA, ClfB, and the fibronectin binding proteins. The mechanism of physically interfering with binding to matrix proteins has also been seen with Pls, a ~230 kDa cell wall associated protein related to SasG that is encoded within the type I SCC*mec* element of some MRSA strains. Pls producing strains have defects in fibronectin binding and cellular invasion that can be mitigated by deleting the *pls* gene [64,65]. This inhibition of fibronectin binding has been attributed to either steric hindrance or competition between Pls and the fibronectin binding proteins for anchoring sites on the cell wall [65]. It has also been observed that under some conditions expression of capsular polysaccharide can interfere with ClfA-mediated binding to fibrinogen, again likely through steric hindrance [66].

Altering expression of large surface proteins also has implications for biofilm formation. Increased biofilm production has been observed previously for both *arlRS* [67,68] and *mgrA* [39,40,58] mutants, and it appears to be polysaccharide independent [39,67]. In agreement with these results, we observed increased biofilm production in the MW2 *mgrA* mutant, which was unaffected by deletion of the *ica* locus encoding the machinery for polysaccharide synthesis (Fig 11). The increased biofilm formation in the *mgrA* mutant appears to be largely due to up-regulation of SasG, a protein known to promote biofilm accumulation [60]. SraP is also reported to contribute to biofilm formation [69], although we did not see a decrease in biofilm biomass when *sraP* was deleted. Deletion of *sasG* in an *mgrA* mutant only partially reduces biofilm production, suggesting that other proteins, such as SasC [46], may be involved. Alternatively, mutation of *mgrA* may promote release of extracellular DNA through increased rates of autolysis, which could contribute to the biofilm matrix. The fact that SasG-producing strains such as the MW2 *mgrA* mutant show both increased biofilm formation and decreased clumping demonstrates that these are different modes of intercellular interaction that are governed by distinct mechanisms.

We show here that regulation of surface proteins like Ebh, SraP, and SasG is likely more important than previously realized, and that production of these proteins can dramatically impact interactions with matrix proteins like fibrinogen. MgrA also regulates various other genes thought to be involved in virulence. For example, it has been suggested that *mgrA* mutants are more susceptible to phagocytosis because they produce less capsular polysaccharide [52,54]. Yet some particularly virulent strains of *S*. *aureus*, including the USA300 lineage, do not produce capsule [70,71]. Alternatively, there are multiple reports that MgrA regulates production of α-toxin, although whether MgrA represses or activates *hla* expression is debated [43,54,72]. In agreement with Gupta *et al*. [54] we did not observe significant changes in *hla* expression in the LAC *mgrA* mutant by RNA-seq. However, we did see an ~20-fold decrease in expression of Panton-Valentine leukocidin (PVL) in the *mgrA* mutant. Several other immune evasion proteins were also down-regulated in the *mgrA* mutant, including the LukAB leukocidin, chemotaxis inhibiting protein CHIP, and staphylococcal complement inhibitor SCIN. Whether these factors contribute to the virulence phenotypes seen with *mgrA* mutants remains to be investigated.

In summary, we have shown that the ArlRS-MgrA regulatory cascade controls expression of a variety of genes, including those for up to eight surface proteins, depending on the strain. Apart from protein A and the fibronectin binding protein FnbB, relatively little is known about these surface proteins, likely because expression of some of them is low in wild type strains *in vitro*. The signal for the ArlRS TCS is still unknown, making it difficult to predict when this system is active. We did identify the first promoter activated by ArlR, the *mgrA* P2 promoter (Fig 4A), which could aid future studies on unraveling the ArlRS regulatory mechanism. Since the ArlRS-MgrA cascade alters the expression of many surface proteins, we would expect to see large changes in behaviors such as clumping, biofilm formation, and adhesion to host tissues. It is possible that expression of these surface proteins is associated with moving into a new environment within the host, or a means to disseminate from a vegetation or abscess community in the later stages of disease. Although there is still much left to learn about the ArlRS-MgrA cascade, it is clear that it is a major switch controlling virulence determinants in *S*. *aureus*, and will likely be a good target for novel therapeutics.

## Materials and Methods

### Ethics statement

The animal studies were reviewed and protocol approved by the University of Iowa Institutional Animal Care and Use Committee. The approved protocol was assigned number 4071100. Animals were anesthetized with the combination of ketamine (10 mg/kg subcutaneously) and xylazine (10 mg/kg subcutaneously). Animals were administered pain relieving medications (buprenorphine; 0.05 mg/kg twice daily subcutaneously) throughout experimentation. Additionally, animals that could not simultaneously maintain upright positions and exhibit normal escape behavior were prematurely euthanized; these criteria are 100% predictive of death in the model used. The University of Iowa is AAALAC accredited, and the centralized facilities meet and adhere to the standards in the “Guide and Care of Laboratory Animals.”

### Reagents and growth conditions

*S*. *aureus* strains and plasmids used in this work are listed in Table 2. For most experiments *S*. *aureus* was cultured in tryptic soy broth (TSB) at 37°C with shaking; for assessment of clumping, strains were grown in brain heart infusion broth (BHI). *E*. *coli* was cultured in lysogeny broth (LB). Antibiotics were added to the media at the following concentrations: chloramphenicol (Cam), 10 μg/mL; erythromycin (Erm), 5 μg/mL; and tetracycline (Tet), 0.625 μg/mL. *E*. *coli* strains with plasmids were maintained on media supplemented with ampicillin at 100 μg/mL; kanamycin, 50 μg/mL; or spectinomycin at 50 μg/mL.

**Table 2:** Strains and plasmids used in this study.

Strain/plasmid	Genotype/properties	Reference
*E*. *coli*		
DH5α	Cloning strain	Protein Express
ER2566	Overexpression strain	NEB
*S*. *aureus*		
RN4220	Restriction deficient cloning host	[73]
AH1263	USA300 CA-MRSA Erm^S^ (LAC*)	[74]
AH3150	LAC* Δ*ebh*	This work
AH3455	LAC* Δ*mgrA*::tetM	This work
AH3481	LAC* Δ*ebh* Δ*mgrA*::tetM	This work
AH3485	LAC* Δ*mgrA*::tetM Φ11::LL29erm *mgrA*	This work
AH3007	LAC* *spa*::ΦNΣ	[33]
AH3488	LAC* *spa*::ΦNΣ Δ*mgrA*::tetM	This work
AH3487	LAC* *spa*::ΦNΣ Δ*mgrA*::tetM Δ*ebh*	This work
AH3052	LAC* Δ*spa*	[75]
AH3056	LAC* Δ*spa* Δ*arlRS*	This work
AH3458	LAC* Δ*spa* Δ*mgrA*::tetM	This work
AH1975	LAC* Δ*arlRS*	[33]
AH3520	LAC* Δ*arlRS*::tetM	This work
AH3577	LAC* Δ*ebh* Δ*mgrA*::tetM *fmtB*::ΦNΣ	This work
AH3738	LAC* Δ*ebh* Δ*mgrA*::tetM *sdrD*::ΦNΣ	This work
AH3739	LAC* Δ*ebh* Δ*mgrA*::tetM *sasG*::ΦNΣ	This work
AH3792	LAC* Δ*ebh* Δ*mgrA*::tetM *sasC*::ΦNΣ	This work
AH3797	LAC* Δ*ebh* Δ*mgrA*::tetM *fnbB*::ΦNΣ	This work
AH3798	LAC* Δ*ebh* Δ*mgrA*::tetM *sraP*::ΦNΣ	This work
AH3808	LAC* *sraP*::ΦNΣ	This work
AH3811	LAC* Δ*mgrA*::tetM *sraP*::ΦNΣ	This work
MW2	USA400 CA-MRSA	[76]
AH3060	MW2 Δ*arlRS*::tetM	This work
AH3456	MW2 Δ*mgrA*::tetM	This work
AH3422	MW2 Δ*mgrA*	This work
AH3494	MW2 Δ*mgrA* Δ*ebh*::tetM	This work
AH3934	MW2 Δ*mgrA* Δ*sraP*	This work
AH3945	MW2 Δ*mgrA* Δ*sraP* Δ*ebh*::tetM	This work
AH3976	MW2 Δ*mgrA* Δ*sraP* Δ*sasG*	This work
AH3977	MW2 Δ*mgrA* Δ*sraP* Δ*sasG* Δ*ebh*::tetM	This work
AH3989	MW2 Δ*mgrA* Δ*sasG*	This work
AH4032	MW2 Δ*mgrA* Δ*sasG* Δ*ebh*::tetM	This work
AH4473	MW2 Δ*mgrA* Δ*ica*::tet	This work
502a		[56]
AH3624	502a Δ*arlRS*::tetM	This work
AH3625	502a Δ*mgrA*::tetM	This work
Newman	MSSA	[77]
AH3062	Newman Δ*arlRS*::tetM	This work
AH3472	Newman Δ*mgrA*::tetM	This work
MRSA252	USA200 HA-MRSA	[78]
AH3608	MRSA252 Δ*arlRS*::tetM	This work
AH3483	MRSA252 Δ*mgrA*::tetM	This work
N315	USA100	[79]
AH3082	N315 Δ*arlRS*::tetM	This work
AH3473	N315 Δ*mgrA*::tetM	This work
MN8	USA200 toxic shock isolate	[80]
AH3063	MN8 Δ*arlRS*::tetM	This work
AH3480	MN8 Δ*mgrA*::tetM	This work
Plasmids		
pJB38	Mutation generation vector, Cam^R^	[81]
pJMB202	*arlRS* deletion vector, Cam^R^	[33]
pHC02	*arlRS*::tetM deletion vector, Cam^R^	This work
pHC12	*ebh* deletion vector, Cam^R^	This work
pHC34	*mgrA* deletion vector, Cam^R^	This work
pHC75	*mgrA*::tetM deletion vector,Cam^R^	This work
pHC76	*sraP* deletion vector, Cam^R^	This work
pHC77	*sasG* deletion vector, Cam^R^	This work
pCM28	*S*. *aureus*–*E*. *coli* shuttle vector, Cam^R^	[74]
pHC66	*mgrA* complementing vector, Cam^R^	This work
pArl	*arlRS* complementing vector, Cam^R^	[33]
pCM29	sGFP expression vector, Cam^R^	[82]
pLL29	*S*. *aureus* integration vector, Tet^R^	[83]
pLL29-erm	*S*. *aureus* integration vector, Erm^R^	This work
pHC67	*mgrA* chromosomal complementing vector, Erm^R^	This work
pCM11	*S*. *aureus* sGFP expression plasmid, Erm^R^	[84]
pCM11-ebh	P_*ebh*_-sGFP fusion, Erm^R^	[33]
pHC68	*mgrA* P2-sGFP fusion, Erm^R^	This work
pHC70	*mgrA* P1-sGFP fusion, Erm^R^	This work
pHC71	*sraP* (P1 and P2)-sGFP fusion, Erm^R^	This work
pHC73	*sraP* P2-sGFP fusion, Erm^R^	This work
pHC07	pET28a-arlR, overexpression, Kan^R^	This work
pHC74	pKLD66-mgrA, overexpression, Amp^R^	This work

Human plasma (HP) was obtained from donors at the University of Iowa Inflammation Program with all necessary approvals. HP was diluted 1:1 with heparin/dextran sulfate to prevent clotting, and for the purposes of this study, this level of HP was considered a final concentration of 100%. Purified human fibrinogen was purchased from Sigma-Aldrich.

### Recombinant DNA and genetic techniques

*E*. *coli* DH5α was used as a cloning host for plasmid constructions. Restriction enzymes, DNA ligase, and Phusion DNA polymerase were purchased from New England Biolabs. The plasmid mini-prep and gel extraction kits were purchased from Invitrogen. Lysostaphin, used for *S*. *aureus* DNA extractions, was purchased from AMBI Products LLC. Plasmids were electroporated into *S*. *aureus* RN4220 as described previously [85]. Bacteriophage transductions between *S*. *aureus* strains were performed with phage 80α or 11 as described previously [86]. All oligonucleotides were ordered from IDT (Coralville, IA) and are listed in S2 Table. DNA sequencing was performed at the University of Iowa DNA Core Facility.

### Clumping assay

Measurements of *S*. *aureus* clumping in the presence of fibrinogen or plasma were performed essentially as described by Walker et al. [33]. Briefly, cultures were grown in BHI to an OD_600_ of 1.5, harvested by centrifugation, and resuspended in the same volume of phosphate buffered saline (PBS). Human plasma was added to a final concentration of 2.5% (vol/vol), and clearing of the suspension was measured over two hours by periodically removing small aliquots from the top of the tube and measuring the OD_600_. Alternatively, clumping was initiated by adding purified fibrinogen to a final concentration of 18.5 μg/mL. Relative clumping values were calculated using the equation % clumping = ((OD_time0_-OD_timeT_)/OD_time0_)x100.

### Preparation of clumps for SEM

The LAC wild type strain, *mgrA*::tet mutant, and chromosomally complemented strain were allowed to clump for 2 hr with fibrinogen as described above. Slides were then prepared for SEM and imaged as described previously [33].

### Immunofluorescence microscopy

Immunofluorescence microscopy was used to visualize Ebh on individual cells. All strains lacked the gene for protein A (*spa*), and contained plasmid pCM29, which constitutively expresses sGFP [82]. Cultures were grown overnight in TSB, washed three times with PBS, and adhered to poly-L-lysine coated glass chamber slides (Nunc). Cells were fixed for 20 min with 4% paraformaldehyde and then washed three times with PBS. Slides were blocked with Superblock (Pierce) containing 5% bovine serum albumin for 30 min and washed three times with PBS. Rabbit anti-Ebh serum [33] was diluted 1:100 in superblock plus 1% BSA and allowed to incubate on slides overnight at 4°C. The slides were then washed five times with Superblock and incubated with a 1:500 dilution of Alexa 568-conjugated goat anti-rabbit antibody (Molecular Probes) for 1 hr at room temperature. Slides were washed an additional five times with PBS, chamber slide wells were removed, and cover slips were mounted with Prolong Diamond Antifade mountant (Molecular Probes). Images were obtained with a Leica DM5500 Q confocal microscope.

### Generation of gene deletions

To construct the *mgrA* deletion plasmid, ~700 bp regions flanking the gene were amplified with primer pairs HC116/HC117 and HC118/119. The products were column purified and fused in a second PCR using primers HC116 and HC119. This fusion product was gel purified, digested with SacI and SalI, and ligated into pJB38 [81] to generate pHC34. This plasmid was electroporated in RN4220, selecting on TSA plates containing Cam at 30°C. The plasmid was then transduced into *S*. *aureus* strains LAC and MW2. Individual colonies were streaked on TSA Cam plates incubated at 42°C to select for integration into the chromosome. Single colonies were grown in TSB at 30°C and diluted 1:500 in fresh media for four successive days before diluting to 10^−6^ and plating on TSA containing 0.2 μg/mL (LAC) or 0.6 μg/mL (MW2) anhydrotetracycline to select for loss of the plasmid. Colonies were screened for resistance to Cam, and Cam^S^ colonies were screened by PCR for deletion of *mgrA*.

The *ebh*, *sraP*, and *sasG* gene deletions were constructed in a similar manner. Sequences flanking *ebh* were amplified with primers HC28/HC53 and HC54/HC55, fused, digested with EcoRI and SalI, and ligated into pJB38 to generate pHC12. For the *sraP* deletion construct, flanking regions were amplified from *S*. *aureus* MW2 genomic DNA using primer pairs HC338/HC339 and HC340/HC341. The fusion product was digested with SacI and SalI and ligated into pJB38 to generate pHC76. The plasmid for deleting *sasG* in MW2 was generated similarly, using primer pairs HC246/HC247 and HC248/HC249. The fusion product was digested with EcoRI and SalI and ligated into pJB38 to generate pHC77.

To generate the *arlRS*::tetM deletion plasmid pHC02, the tetracycline resistance cassette was amplified from pTET [87] using primers HC3 and HC4. The resulting product was digested with NheI and ligated into the NheI site located between the *arlRS* flanking segments in pJMB202. Likewise, the *mgrA*::tetM deletion vector pHC75 was constructed by ligating the same tetracycline resistance cassette into the NheI site of pHC34.

### Construction of the *mgrA* chromosomal complement

The *S*. *aureus* chromosomal integration vector pLL29 [83], which confers resistance to tetracycline, was modified to generate pLL29erm, an erythromycin resistant variant. The *ermC* gene was amplified from pCM11 [84] using primers HC156 and HC157, and the resulting product was digested with BsrGI and NheI. pLL29 was digested with the same enzymes to remove the *tetK* gene, and the *ermC* cassette was ligated in its place. The ligation reaction was transformed into *E*. *coli* DH5α, selecting for spectinomycin resistant colonies. To generate pHC67 (pLL29erm *mgrA*), a 910-bp fragment containing the *mgrA* gene and both of its promoters was amplified using primers HC148 and HC169. The product was digested with BamHI and HindIII and ligated into the same sites in pLL29erm. This plasmid was electroporated into RN4220 containing the helper plasmid pLL2787 [83], and integration into the chromosome was confirmed by PCR using primer sets HC172/scv10 and scv8/scv9. The integrated construct was then transduced into the LAC Δ*mgrA*::tet strain, selecting for Erm resistance.

### Construction of the *mgrA* complementing vector

The same 910-bp fragment described above, containing the *mgrA* gene and its native promoters, was amplified from LAC chromosomal DNA using primers HC169 and HC187. The product was digested with BamHI and SalI and ligated into the same sites in pCM28 [74] to generate pHC66. This plasmid was electroporated into RN4220 and subsequently transduced into LAC Δ*mgrA*::tet and LAC Δ*arlRS*::tet.

### *mgrA* and *sraP* promoter fusions

All promoter-sGFP transcriptional reporters were generated in the shuttle vector pCM11 [84]. We generated transcriptional reporters for each of the *mgrA* promoters separately, based on the *mgrA* promoter mapping reported by Ingavale *et al*. [38]. A fragment containing the upstream promoter, P2, was amplified from LAC genomic DNA using primers HC184 and HC191. Likewise, a fragment containing only the downstream promoter, P1, was amplified using primers HC185 and HC194. The PCR products were digested with HindIII and KpnI, and subsequently ligated upstream of the sGFP gene in pCM11, to generate plasmids HC68 and HC70, respectively.

The *sraP*-sGFP transcriptional fusions were generated by amplifying fragments of increasing length from the region upstream of the *sraP* gene and cloning them into pCM11. To construct pHC73, a 143-bp fragment upstream of the *sraP* start codon was amplified with primers HC290 and HC291, digested with HindIII and KpnI, and ligated into pCM11. pHC71 was constructed in a similar fashion, except that primers HC288 and HC291 were used to amplify a 518-bp fragment that was then cloned into pCM11. All transcriptional fusion plasmids were electroporated into RN4220 and subsequently transduced into the LAC strains of interest.

To assess expression, overnight cultures were diluted 1:100 in TSB in a black 96-well plate, and plates were incubated at 37°C with shaking in a humidified microtiter plate shaker (Stuart). A Tecan Infinite M200 plate reader was used to periodically measure OD_600_ and fluorescence intensity with excitation at 495 nm and emission at 515 nm. Values from quadruplicate wells were averaged, and the experiment was repeated at least once.

### RNA purification

Cultures were grown in TSB to an OD_600_ of 1.5, at which point cells were pelleted and washed briefly with RNAprotect Bacterial Reagent (Qiagen). Cells were lysed with lysostaphin for 1 h at room temperature, and RNA was purified using the RNeasy Mini Kit (Qiagen). Following purification, genomic DNA was removed using the Turbo DNA free kit (Ambion).

### Quantitative polymerase chain reaction

DNase-treated RNA was used as a template to generate cDNA with the High-Capacity Reverse Transcription Kit (Applied Biosystems). Primers for *coa*, *sak*, *vWbp*, *srtA*, *clfA* and *ebh* have been described previously [33]. Primers specific for the *sraP* gene cluster were designed using the PrimerQuest tool on the IDT website (see S2 Table for primer sequences). qPCR was performed by amplifying 10 ng of cDNA with Power SYBR Green Master Mix (Applied Biosystems) under the following conditions: 10 minutes at 95°C, 40 cycles of 15 seconds at 95°C and 1 minute at either 53°C (*coa*, *sak*, *vWbp*, *srtA*, *clfA*, and *ebh* primers) or 57°C (*sraP* gene cluster), followed by a dissociation curve. Expression was normalized to that of DNA gyrase (*gyrB*), and values represent averages of three biological replicates.

### Promoter mapping using 5’RACE

Mapping of the *ebh* and *sraP* promoters using rapid amplification of 5’ cDNA ends (5’ RACE) was performed as described [88]. Template RNA was obtained from an LAC *arlRS* mutant to identify the *ebh* promoter, and from an *mgrA* mutant for the *sraP* promoters. Gene specific primers are listed in S2 Table, and reactions were performed using SuperScript III reverse transcriptase (Invitrogen Life Technologies) and terminal transferase (New England Biolabs).

### RNA-seq

RNA was prepared from triplicate cultures of LAC and LAC Δ*mgrA*::tet, treated with DNase, and assessed for quality using a Bioanalyzer (Agilent). rRNA was depleted using the Ribo-Zero rRNA Removal Kit for Gram-positive bacteria (Illumina). cDNA libraries were generated at the University of Iowa Genomics Division using the TruSeq Stranded mRNA Library Prep Kit (Illumina). Samples were barcoded, pooled, and sequenced in 100x100 paired end reads using a HiSeq 2000 sequencer (Illumina). The resulting sequences were aligned to the USA300_FPR3757 genome sequence using SeqMan NGen (DNASTAR) and the alignment data were analyzed using ArrayStar (DNASTAR). Genes were considered differentially expressed if they showed a ≥4-fold change in expression with 95% confidence as evaluated using the student’s t-test with a false discovery rate (FDR) correction applied for multiple t-tests.

### Cloning and purification of ArlR and MgrA

The *arlR* gene was amplified from LAC genomic DNA using primers HC41 and HC42. The PCR product was digested with NdeI and Xho, and ligated into pET28a to generate pHC07, which expresses *arlR* with an N-terminal His_6_ tag. pHC07 was transformed into the *E*. *coli* overexpression strain ER2566. Cells were grown in LB supplemented with kanamycin at 37°C to an OD_600_ of 0.6, at which point expression was induced by adding 0.1 mM IPTG and shifting to 30°C overnight. Cells were harvested by centrifugation and stored at -20°C. To purify His_6_-ArlR, cells were resuspended in bind buffer (50 mM Na phosphate, 300 mM NaCl, pH 8) and lysed by two passages through a Microfluidics LV1. Cell debris was removed by centrifugation and the soluble fraction was passed over a HIS-Select nickel affinity (Sigma-Aldrich) column. The column was washed with bind buffer supplemented with 10 mM imidazole, and purified ArlR was eluted with elute buffer (50 mM Na phosphate, 300 mM NaCl, 250 mM imidazole, pH 8). Fractions containing ArlR were pooled and dialyzed twice against storage buffer (50 mM Na phosphate, 150 mM NaCl, pH 8), with 0.5 mM EDTA added to the first dialysis buffer. The dialyzed protein was concentrated and glycerol was added to 20% (vol/vol) before freezing in an ethanol/dry ice bath and storage at -80°C.

The *mgrA* coding sequence was amplified from LAC genomic DNA using primers HC148 and HC190. The product was digested with KpnI and HindIII and ligated into the overexpression vector pKLD66 [89] to generate pHC74. This plasmid expresses *mgrA* with sequential His_6_ and maltose binding protein tags at the N-terminus, both of which can be removed by cleavage with Tev protease. pHC74 was transformed into *E*. *coli* expression strain ER2566. For overexpression of *mgrA*, cells were grown with shaking at 37°C in LB supplemented with ampicillin. When the OD_600_ reached ~0.5, expression was induced with 0.5 mM IPTG and the culture was shifted to 30°C overnight. Cells were harvested by centrifugation and the pellet was stored at -80°C. To purify tagged MgrA, the cell pellet was resuspended in bind buffer and cells were lysed by adding lysozyme and sonicating in four ~1 min pulses at 50% duty. Tagged MgrA was purified as described above for ArlR. Fractions containing MgrA were pooled and incubated with a 1:20 molar ratio of His_6_-Tev protease [90] to MgrA at room temperature for 3 h, while dialyzing against bind buffer containing 0.5 mM EDTA. The cleaved protein was dialyzed two additional times against bind buffer at 4°C. To purify MgrA away from the His_6_-MBP tag and His_6_-Tev protease, the cleaved protein was passed over the nickel affinity column again, where MgrA eluted in the flow-through and wash steps. Purified, untagged MgrA was dialyzed against storage buffer and frozen as described for ArlR.

### Western and dot blots

All Western and dot blots were performed using strains lacking protein A (*spa*). Ebh production was assessed by dot blot using antibodies raised against the H2 peptide within the G/A repeat region of Ebh as previously described [33]. Antibodies specific for ArlR and MgrA were generated in rabbits using the purified proteins described above.

To monitor MgrA and ArlR protein production, cultures were diluted 1:100 in TSB and aliquots were removed at each time point. The OD_600_ was measured, and 1 OD_600_ unit of cells was centrifuged, washed once with tris-buffered saline (TBS), and frozen at -20°C. Cell pellets were resuspended in 200 μL TBS and incubated for 1 h at 37°C with 5 μg lysostaphin and 1 U DNase (New England Biolabs). Cell debris was removed by centrifugation and the soluble fraction was heated to 95°C before loading on a 15% SDS-PAGE gel. Proteins were separated by electrophoresis and transferred to a nitrocellulose membrane. The membrane was blocked for 1 h at room temperature with TBS containing 0.05% Tween 20 (TBST) and 5% milk, and then incubated for 1 hr with ArlR or MgrA antiserum (diluted 1:1000 in TBST + 5% milk). The membrane was washed three times with TBST and incubated with HRP-conjugated goat anti-rabbit antibodies (diluted 1:20,000 in TBST + 5% milk). The membrane was washed three times in TBST before incubation with SuperSignal West Pico chemiluminescent substrate for 5 min and exposure to X-ray film. Band intensities were quantified using Image Studio Lite (LI-COR). Results are representative of two (ArlR) or three (MgrA) separate experiments.

### Electrophoretic mobility shift assays

MgrA binding to the *ebh* promoter was observed using a probe labeled at one end with IRDye 700 (LI-COR), synthesized by Integrated DNA Technologies. 50-mer oligos HC487 and HC489 were combined in PBS + 1 mM EDTA and annealed by heating to 95°C for 5 min and then gradually cooling by 1°C per minute to 25°C. Three additional unlabeled probes were prepared in a similar fashion: a specific competitor with the same sequence (primers HC488/HC489), a competitor in which the putative MgrA binding site had been mutated (primers HC492/HC493), and a non-specific competitor from within the *sraP* coding sequence (primers HC349/HC350). For *sraP* P2 EMSAs an IRDye 700 labeled probe was generated with 50-mer oligos HC336 and HC346. An unlabeled specific competitor probe with the same sequence was generated with oligos HC336 and HC337, and a competitor in which the putative MgrA binding site had been mutated was made with oligos HC347 and HC348. The nonspecific competitor probe was the same as for the *ebh* EMSA described above. Binding reactions contained 50 nM labeled probe, 500 nM competitor probe, 25 mM HEPES pH 7.4, 50 mM KCl, 1 mM DTT, and MgrA (0–2 μM). Reactions were incubated for 20 min at room temperature, and then separated on a pre-run 5% TBE-acrylamide gel at 100 V for 1 h in the dark at 4°C. Images were obtained using an Odyssey CLx imaging system (LI-COR).

### Biofilm assay

*S*. *aureus* overnight cultures were diluted 1:40 in BHI supplemented with 0.25% glucose in a 48-well polystyrene microtiter plate. The plate was incubated statically at 37°C in a humidified plate incubator (Stuart) for 16 h. At this point the media was removed and remaining biomass was stained with 0.1% crystal violet. The wells were then washed with sterile distilled water and the plate was photographed with a GelDoc XR+ (Biorad). For quantification, the crystal violet stain was resuspended with isopropanol and the absorbance was measured at 595 nm.

### Infective endocarditis and sepsis

New Zealand white rabbits (approximately 2–3 kg), either sex, were purchased from Bakkom Rabbitry, Red Wing, MN and used according to University of Iowa IACUC approved protocol 4071100. Rabbits were anesthetized with ketamine (25 mg/kg) and xylazine (25 mg/kg) (Phoenix Pharmaceuticals, Burlingame, CA). Their necks were shaved, and 5 cm incisions were made to expose the left carotid arteries. Hard plastic catheters were inserted into the carotid arteries until the catheters just abutted against the aortic valves. The catheters were then tied in place and allowed to cause damage to the aortic valves for 2 h. Subsequently, the catheters were removed and carotid arteries tied off, and the animals were closed. Animals were injected intravenously through the marginal ear veins with *S*. *aureus* strains in 1 ml PBS (approximately 2.5 x 10^7^ CFU/ml for MW2; 1.3 x 10^8^ CFU/ml for 502a). The rabbits were monitored for health status for up to 4 days; during this time, animals that simultaneously failed to exhibit escape behavior and failed to be able to right themselves, 100% predictive of lethal infection, were prematurely euthanized with 1 ml/kg of Beuthanasia D (Shering-Plough, Westlake, TX). After 4 days (or at the time of premature euthanasia), the animals were euthanized, hearts removed, and vegetation formation determined. Vegetations, cauliflower-like clumps of bacteria and host cells, were removed, weighed, and homogenized for CFU determination. Statistical differences in vegetation weights and CFUs were determined by Student’s *t* test analysis of normally-distributed, non-paired data. Differences in animal survival rates were determined by log-rank (Mantel-Cox) test.

## Supporting Information

S1 Fig Clumping of LAC *clfA clfB fnbAB* mutant strains. Clumping of LAC *clfA clfB fnbAB* mutants in the presence of human plasma (A) or purified human fibrinogen (B). Values represent averages and standard deviations of three separate experiments.
(PDF)

S1 Table Genes regulated by MgrA. (PDF)

S2 Table Primers used in this work. (PDF)
