# Supplementary material for: The Staphylococcus aureus Global Regulator MgrA Modulates Clumping and Virulence by Controlling Surface Protein Expression
Source: PLoS Pathog. 2016 May 4;12(5):e1005604. doi: 10.1371/journal.ppat.1005604 (PMC4856396; doi:10.1371/journal.ppat.1005604)
Supplement: S1 Fig — Clumping of LAC clfA clfB fnbAB mutants in the presence of human plasma (A) or purified human fibrinogen (B). Values represent averages and standard deviations of three separate experiments. (PDF) [file ppat.1005604.s001.pdf]

Supplementary Figure 1

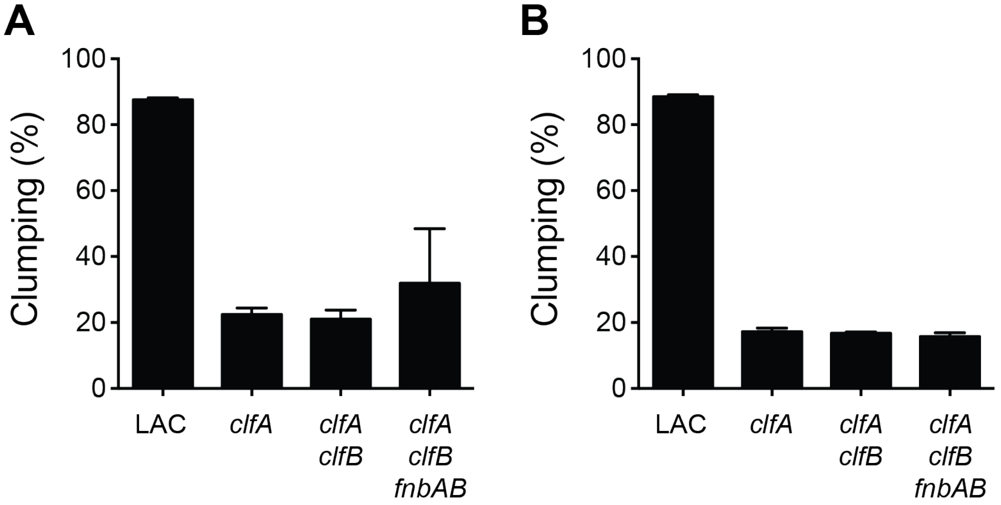

Fig. S1 Clumping of LAC *clfA clfB fnbAB* mutants in the presence of human plasma (A) or purified human fibrinogen (B). Values represent averages and standard deviations of three separate experiments.
