# Supplementary material for: The Staphylococcus aureus Global Regulator MgrA Modulates Clumping and Virulence by Controlling Surface Protein Expression
Source: PLoS Pathog. 2016 May 4;12(5):e1005604. doi: 10.1371/journal.ppat.1005604 (PMC4856396; doi:10.1371/journal.ppat.1005604)
Supplement: S1 Table — (PDF) [file ppat.1005604.s002.pdf]

Table S1. Genes up ≥4-fold in *mgrA* mutant

SAUSA300 orf number	Fold change	Gene name	Function
0113	22.3	*spa*	immunoglobulin G binding protein A
0130	97.1		NAD-dependent epimerase
0131	106.5		putative sugar transferase
0132	48.2		glycosyl transferase
0133	25.0		hypothetical protein
0134	7.6		polysaccharide extrusion protein
0346	4.0		high affinity iron transporter
0547	20.0	*sdrD*	serine-aspartate repeat-containing protein SdrD
0577	4.7		transcriptional regulator
0954	43.9	*atlR*	MarR family transcriptional regulator
1005	22.8	*mntH*	manganese transporter
1140	165.1	*lytN*	peptidoglycan hydrolase
1141	91.0	*fmhC*	endopeptidase resistance protein
1142	14.4	*dprA*	DNA protecting protein
1202	8.0		XRE-family transcriptional regulator
1214	4.8		putative threonine aldolase
1294	5.8		hypothetical protein
1327	44.7	*ebh*	cell surface protein
1702	7.7	*sasC*	cell surface protein
1739	4.4		hypothetical protein
1740	4.6		putative permease
1741	4.7		putative lipoprotein
2109	24.5	*fmtB*	truncated FmtB protein
2110	41.4	*fmtB*	truncated FmtB protein
2218	10.3	*sarV*	MarR family transcriptional regulator
2237	8.6		putative urea transporter
2238	74.3	*ureA*	urease subunit gamma
2239	61.5	*ureB*	urease subunit beta
2240	33.7	*ureC*	urease subunit alpha
2241	22.5	*ureE*	urease accessory protein UreE
2242	20.2	*ureF*	urease accessory protein UreF
2243	14.9	*ureG*	urease accessory protein UreG
2244	13.0	*ureD*	urease accessory protein UreD
2435	7.5	*sasG*	truncated surface protein G
2436	8.3	*sasG*	truncated surface protein G
2440	5.4	*fnbB*	fibronectin binding protein B
2463	17.2	*ddh*	D-lactate dehydrogenase
2465	21.5		putative peptide transport system ATP-binding protein
2466	8.6		putative peptide transport system permease protein
2488	6.9	*feoA*	ferrous iron transport protein A
2522	4.6		hypothetical protein
2523	4.4		hypothetical protein
2524	4.2		hypothetical protein
2555	10.7		glutathione peroxidase
2585	4.7	*asp3*	accessory secretory protein Asp3
2586	5.1	*asp2*	accessory secretory protein Asp2
2587	5.5	*asp1*	accessory secretory protein Asp1
2588	5.2	*secY2*	preprotein translocase subunit SecY2
2589	5.0	*sraP*	serine-rich adhesin for platelets
2596	13.8	*cap1C*	capsular polysaccharide biosynthesis protein Cap1C

2597	18.9	*cap1B*	capsular polysaccharide biosynthesis protein Cap1B
2598	18.5	*cap1A*	capsular polysaccharide biosynthesis protein Cap1A
2612	4.2	*hisG*	ATP phosphoribosyltransferase catalytic subunit
2613	5.2	*hisZ*	ATP phosphoribosyltransferase regulatory subunit
2614	7.7		putative polysaccharide deacetylase

Genes down ≥4-fold in *mgrA* mutant

SAUSA300 orf number	Fold change	Gene name	Function
0067	5.1		universal stress protein
0151	5.7	*adhE*	bifunctional acetaldehyde-CoA/alcohol dehydrogenase
0152	8.7	*cap5A*	capsular polysaccharide biosynthesis protein Cap5A
0153	4.0	*cap5B*	capsular polysaccharide biosynthesis protein Cap5B
0154	5.0	*cap5C*	capsular polysaccharide biosynthesis protein Cap5C
0156	4.2	*cap5E*	capsular polysaccharide biosynthesis protein Cap5E
0174	4.7		hypothetical protein
0175	4.8		putative lipoprotein
0176	5.2		ABC transporter permease
0177	6.1		hypothetical protein
0178	4.4		hypothetical protein
0220	4.2	*pflB*	formate acetyltransferase
0221	4.9	*pflA*	pyruvate formate-lyase activating enzyme
0382	4.1		sodium:dicarboxylate symporter family protein
0491	5.5	*cysK*	cysteine synthase A
0594	5.1	*adh*	alcohol dehydrogenase
0667	4.5		hypothetical protein
0672	3985	*mgrA*	transcriptional regulator
0673	5.7		cobalamin synthesis protein/P47K family protein
0776	5.0	*nuc*	nuclease
0815	8.9	*ear*	Ear protein
1295	17.1		CSD family cold shock protein
1329	4.2		amino acid permease
1330	4.3	*ilvA*	threonine dehydratase
1331	4.1	*ald*	alanine dehydrogenase
1381	19.2	*lukF*	Panton-Valentine leukocidin, LukF-PV
1382	23.4	*lukS*	Panton-Valentine leukocidin, LukS-PV
1440	5.8		hypothetical protein
1581	7.5		hypothetical protein
1582	4.4		hypothetical protein
1678	4.7	*fhs*	formate-tetrahydrofolate ligase
1753	8.7	*splF*	serine protease SplF
1754	7.1	*splE*	serine protease SplE
1755	8.4	*splD*	serine protease SplD
1756	8.0	*splC*	serine protease SplC
1757	10.2	*splB*	serine protease SplB
1758	10.2	*splA*	serine protease SplA
1918	5.9		truncated beta-hemolysin
1919	4.9		staphylococcal complement inhibitor SCIN
1920	16.0	*chs*	chemotaxis-inhibiting protein CHIP
1974	7.9	*lukB*	leukocidin
1975	7.5	*lukA*	leukocidingr
2164	4.5		hypothetical protein

2245	4.8	*sarR*	SarR transcriptional regulator
2453	5.8		ABC transporter ATP-binding protein
2454	6.1		membrane spanning protein
2550	5.7	*nrdG*	anaerobic ribonucleotide reductase, small subunit
2551	4.5	*nrdD*	anaerobic ribonucleoside triphosphate reductase
2572	6.9	*aur*	zinc metalloproteinase aureolysin
