# Supplementary material for: The Staphylococcus aureus Global Regulator MgrA Modulates Clumping and Virulence by Controlling Surface Protein Expression
Source: PLoS Pathog. 2016 May 4;12(5):e1005604. doi: 10.1371/journal.ppat.1005604 (PMC4856396; doi:10.1371/journal.ppat.1005604)
Supplement: S2 Table — (PDF) [file ppat.1005604.s003.pdf]

Table S2. Primers used in this work

Code	Name	Sequence
HC3	TetM 5'NheI	GTTAGCTAGCCCTAGGCAAATATGCTCTTACGTGC
HC4	TetM 3'NheI	GGCATGCTAGCGCACTAAGTTATTTTATTGAACATATATCTTAC
HC28	Ebh_N_delA_EcoRI	GCATGAATTCGCGAAATTGTATTGAATCGTCA
HC41	ArlR_5NdeI	GTACATATGATGACGCAAATTTTAATAGTAGAAGATGAAC
HC42	ArlR_3XhoI	GATCTCGAGTCATCGTATCACATACCCAACG
HC53	Ebh delB MCS	ACGCGTGGTACCGCTAGCATGTTGTACGTTACCATTATCAGATG
HC54	Ebh delC MCS	GCTAGCGGTACCACGCGTTTGAACAATGGTATTGCTCGAATTTC
HC55	Ebh delD SalI	GTTCGTCGACTGA TGC TGA CTT TTT GTC TTT GGA T
HC62	Ebh GSP-RT	CAT GAG TAG CAG ATA ATG ATT GAC CAT
HC63	Ebh GSP1	GTA CTT GAG ATT CTC GAT TCT CAG
HC64	Ebh GSP2	TAA CCA CGC TTG CTG GTT GAT
HC116	MgrA delA SacI	GAC GAGCTC TCACTTAGTTTCAACTGATCTTCCA
HC117	MgrA delB	CTC GAG GGT ACC GCT AGC AGA CAT TAA AGT TCT CCT CCA GAC
HC118	MgrA delC	GCT AGC GGT ACC CTC GAG TAA TTA ACT TTT GTC ATG ACA ATT AAA GTA ATG
HC119	MgrA delD SalI	CGT GTC GAC GGTCCTTCTGGTTCAGGAAA
HC148	MgrA down 3'HindIII	CCC AAGCTT TTA TTT TTC CTT TGT TTC ATC AAA TGC ATG
HC156	ErmC +promoter 5'BsrGI	GTTGTT TGTACA CAATAATCGCATCCGATTGCAGTAT
HC157	ErmC 3'NheI	GTTGTT GCT AGC CGA TTC ACA AAA AAT AGG CAC ACG
HC169	MgrA prom3 5'BamHI	CTC GGATCC GACAAGTTCTTGTTTAAGATCGCAAC
HC172	scv4-erm	GAGCCGATTTCAAAGATATTATCATGTTC
HC184	PmgrA v2 5'HindIII	AAAAAGCTTCGTCCCCTTTTAAAGCAATG
HC185	PmgrA v3 5'HindIII	AAAAAGCTTAACGTGGTTTTACCACTACCCAAA
HC187	MgrA 3'SalI	CCC GTCGAC TTA TTT TTC CTT TGT TTC ATC AAA TGC ATG
HC190	MgrA 5'KpnI	GTA GGTACC ATGTCTGATCAACATAATTTAAAAGAACAGC
HC191	PmgrA2 3'KpnI	AAGGTACC AAT AAG AAT ATC CAT AAT TAA CGG ATT TTT GG
HC194	PmgrA3 3'KpnI	AAGGTACCTCC GTT TTT TTC TCT TTT CGG ATT G
HC197	ebh int fwd	TCGGTTGAGTCAAGAGCTG
HC198	ebh int rev	GATAGCCCGAACCATCATTA
HC246	MW2sasG delA EcoRI	ATGGAATTCAATGATTTGAAAAGCAAGAGCAATA
HC247	MW2sasG delB	CTCGAGGGTACCGCTAGCATCTCTCATTTGCATACTCCTTTTTCC
HC248	MW2 sasG delC	GCTAGCGGTACCCTCGAGGCTGGATTAATGTTATTGGCACGT
HC249	MW2sasG delD SalI	GAT GTC GAC TTGATGTTATTGCAAGTAAAGGAAT
HC288	PsraP v1 5'HindIII	GAAAAGCTTGTG AAT TAA ATG CAT CCT TAC TAA AC
HC290	PsraP v3 5'HindIII	GAAAAGCTTATA TGA AAA CGA TTA CAT TTA GAC GAA
HC291	PsraP 3'KpnI	AAGGTACC CCT TGT ATT ATC TTT ACA TTC ATT AGA TTA TA
HC294	sraP GSP-RT	CCG TAG TTT TCA GTC CGT ATC C
HC295	sraP GSP1	CAC TAA ACT ATG ACT AAT AAA TGG TAG CC
HC296	sraP GSP2	TTCTTTAATTCCGGATTTTACCCAATT
HC336	sraP probe5 fwd	TTTGTATCAAAAAAGAGTTGTATTAAATTGAATAGTGGCATATATTATAA
HC337	sraP probe5 rev	TTATAATATATGCCACTATTCAATTTAATACAACTCTTTTTTGATACAAA
HC338	MW2 sraP delA SacI	GACGAGCTCACGATTACACAGTAGTGAATGAGAT
HC339	MW2 sraP delB	CTCGAGGGTACCGCTAGCCCAGATTTATAAAGTCTTACTCTTGTTT
HC340	MW2 sraP delC	GCTAGCGGTACCCTCGAGTGGCGTTATGACATTATTAGTTGGT

HC341	MgrA delD SalI	CGT GTC GAC CAT TAC AAT CGG CAT TGG TCC TG
HC346	sraP probe5 rev IRD700	TTATAATATATGCCACTATTCAATTTAATACAACTCTTTTTGATACAAA
HC347	sraP probe5 mut fwd	TTTGTAGATGTCAAGCACCTCATTAAATTGAATAGTGGCATATATTATAA
HC348	sraP probe5 mut rev	TTA TAA TAT ATG CCA CTA TTC AAT TTA ATG AGG TGC TTG ACA TCT ACA AA
HC349	sraP probe 6 fwd	CTGGAAAAAATTGGGTAAAATCCGGAATTAAAGAAATAGAAATGTTCAAA
HC350	sraP probe 6 rev	TTTGAACATTTCTATTTCTTTAATTCCGGATTTTACCCAATTTTTTCCAG
HC487	ebh probe2 fwd IRD700	GAGCATAAATAAATTGTTCAACACATAGTTGTAATGTGTTTCAATACTTT
HC488	ebh probe2 fwd comp	GAGCATAAATAAATTGTTCAACACATAGTTGTAATGTGTTTCAATACTTT
HC489	ebh probe2 rev	AAAGTATTGAAACACATTACAACTATGTGTTGAACAATTTATTTATGCTC
HC492	ebh probe 2 mut fwd	GAGCATAAATAAATTGTTTGTAACATCAGAATAATGTGTTTCAATACTTT
HC493	ebh probe 2 mut rev	AAAGTATTGAAACACATTATTCTGATGTTACAAACAATTTATTTATGCTC
qRT-PCR primers		
HC314	sraP 1738 for	ACTGTAGGCAATCAAACCATAGA
HC315	sraP 1851 rev	CCG CTT GGT AAT CCT GTA ACT
HC318	secY2 367 for	GTTTCGAAAGAACGTGTTCATCA
HC319	secY2 479 rev	CGA TGC CGT AGC GAC TAT TT
HC320	asp1 876 for	GAAGTATCAGCGGGAGAATACAA
HC321	asp1 1001 rev	CAG CGT CAC TCA TAC CAT CTA TC
HC322	asp2 734 for	GGAACGGGACGGTACATTTAG
HC323	asp2 824 rev	CTA CGA AAC GAC TAC CAC CAA G
HC324	asp3 521 for	CAGAACCATCTGACACCTATCC
HC325	asp3 620 rev	CCT CGA TAT TGT CGT CCA TCC
HC467	secA2 208 for	GAAGCGAGCTGGAGAGTATTAG
HC468	secA2 303 rev	GTTTGCATCTCCGCAATGTTA
HC328	gtfA 1240 for	GGCTCAGGACTAGGTATGATTG
HC329	gtfA 1352 rev	CCA CAC TAT CTT CGT CGG TAT C
HC330	gtfB 589 for	ATTAACTCACTAGCCCTACCATTT
HC331	gtfB 692 rev	TTC CCG GGA CAT TAC CAT TAC
